# Recent Advancements in Emerging Technologies for Healthcare Management Systems: A Survey

**DOI:** 10.3390/healthcare10101940

**Published:** 2022-10-03

**Authors:** Sahalu Balarabe Junaid, Abdullahi Abubakar Imam, Abdullateef Oluwagbemiga Balogun, Liyanage Chandratilak De Silva, Yusuf Alhaji Surakat, Ganesh Kumar, Muhammad Abdulkarim, Aliyu Nuhu Shuaibu, Aliyu Garba, Yusra Sahalu, Abdullahi Mohammed, Tanko Yahaya Mohammed, Bashir Abubakar Abdulkadir, Abdallah Alkali Abba, Nana Aliyu Iliyasu Kakumi, Saipunidzam Mahamad

**Affiliations:** 1 Department of Computer Science, Ahmadu Bello University, Zaria 810211, Nigeria; 2 School of Digital Science, Universiti Brunei Darussalam, Brunei Darussalam, Jalan Tungku Link, Gadong BE1410, Brunei; 3 Department of Computer Science, University of Ilorin, Ilorin 1515, Nigeria; 4 Department of Computer and Information Science, Universiti Teknologi PETRONAS, Sri Iskandar 32610, Malaysia; 5 Department of Electrical Engineering, University of Jos, Bauchi Road, Jos 930105, Nigeria; 6 SEHA Abu Dhabi Health Services Co., Abu Dhabi 109090, United Arab Emirates; 7 Department of Chemistry, Gombe State University, Gombe 760253, Nigeria; 8 Patient Care Department, General Ward, Saudi German Hospital Cairo, Taha Hussein Rd, Huckstep, El Nozha, Cairo Governorate 4473303, Egypt

**Keywords:** wearables, sensors, internet of things, artificial intelligence, blockchain, healthcare

## Abstract

In recent times, the growth of the Internet of Things (IoT), artificial intelligence (AI), and Blockchain technologies have quickly gained pace as a new study niche in numerous collegiate and industrial sectors, notably in the healthcare sector. Recent advancements in healthcare delivery have given many patients access to advanced personalized healthcare, which has improved their well-being. The subsequent phase in healthcare is to seamlessly consolidate these emerging technologies such as IoT-assisted wearable sensor devices, AI, and Blockchain collectively. Surprisingly, owing to the rapid use of smart wearable sensors, IoT and AI-enabled technology are shifting healthcare from a conventional hub-based system to a more personalized healthcare management system (HMS). However, implementing smart sensors, advanced IoT, AI, and Blockchain technologies synchronously in HMS remains a significant challenge. Prominent and reoccurring issues such as scarcity of cost-effective and accurate smart medical sensors, unstandardized IoT system architectures, heterogeneity of connected wearable devices, the multidimensionality of data generated, and high demand for interoperability are vivid problems affecting the advancement of HMS. Hence, this survey paper presents a detailed evaluation of the application of these emerging technologies (Smart Sensor, IoT, AI, Blockchain) in HMS to better understand the progress thus far. Specifically, current studies and findings on the deployment of these emerging technologies in healthcare are investigated, as well as key enabling factors, noteworthy use cases, and successful deployments. This survey also examined essential issues that are frequently encountered by IoT-assisted wearable sensor systems, AI, and Blockchain, as well as the critical concerns that must be addressed to enhance the application of these emerging technologies in the HMS.

## 1. Introduction

The advancement of information technology (IT) has resulted in significant improvements in health care services, particularly in remote health monitoring [1]. One of the primary purposes of employing physical sensor networks is to focus on disease prevention and early identification of high-risk disease disabilities [2]. Today, smart technologies and sophisticated instruments (such as smart wireless and wearable sensors) have substantially risen for rapid monitoring and control of patients’ situations via prompt access and continuous assessment of patients’ vital health signs [3].

The capacity of such smart devices to store and transport data is critical in several forms of healthcare or medical care (for example, telemedicine) [4]. Wearable sensors are primarily used to observe and track patients’ health problems and status, and a variety of other health-related functionalities [1,2]. In other words, the vital health signs represent the patient’s physiological status, organ activity, and illness progression. The assessment of these indicators has a significant influence on disease prevention, diagnosis, treatment, and nursing care [5]. These health data, if assessed accurately and promptly, might provide a useful reference for efficient and high-quality medical care. Many smart devices, Internet of Things (IoT), and artificial intelligence (AI)-based technologies have been designed and developed to enhance prompt and continuous assessment of patient’s health status and applicable healthcare sub-systems.

Smart devices, specifically wearable sensors, have attracted a lot of attention in the last decade, mostly in the healthcare field. Such devices seek to derive therapeutically important health-related data from physical (body) indicators such as heart rate (HR), blood pressure (BP), body temperature, respiration rate, and body motion [6]. That is, basic health information is derived and shared using applicable wearable sensors and wearable sensor networks. Wearable sensor networks (WSNs) are made up of a variety of health-related sensors [2]. Such networks’ sensors are put on various regions of the body, and these sensors may be worn or implanted on the patient’s body. Each of these sensors has unique criteria for identifying and recording symptoms (health-related data) [2,5,6]. However, due to many diseases and impairments, patient monitoring continuity for prompt medical intervention and delivery is pivotal [2,3]. As a result, using WSNs to monitor patients is a key area of deployment of smart wearable technology in the healthcare domain.

Furthermore, the successful alliance of AI and healthcare has morphed into improved patient healthcare in areas ranging from hospital productivity [7] and patient safety [8] to quality medical treatment [9]. AI as a tool and/or technology is used to analyze and visualize patient data for adequate healthcare administration [10]. Much of the research on the influence of AI on medical outcomes has been beneficial and encouraging [11]. For example, health professionals and patients are increasingly utilizing and managing medical applications and medical-based games [12] not only to remotely monitor patients but also as evidence-based medicine [13]. This phenomenon is observed in both doctors and patients alike. The adoption of AI in healthcare gives credence to patient empowerment and a more equitable dialogue between doctors and patients. A practical example is the use of cloud computing with AI to enhance access to health data and the administration of medical resources [3,14]. In terms of data, patients’ health data are required to tailor specific patient treatment and it can be further utilized for disease prediction and healthcare policymaking through big data analytics (BDA) [15]. The IoT as a tool can be paired with AI-based technologies or platforms to further improve and promote quality healthcare delivery [16]. The success of IoT in various application domains serve as indicator for its acceptance and integration with wearable sensors and AI technologies for quality healthcare delivery. Wearable sensors are used as objects or components in IoT and are controlled via the communication links such as Bluetooth, Wi-Fi and in recent time, the Internet.

**Figure 1:**
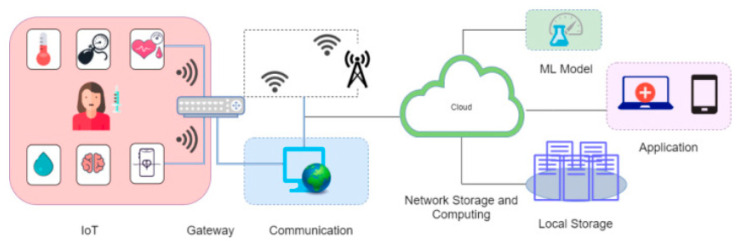
A high-level illustration of the IoT-ML-powered healthcare delivery system [21].

With the introduction of IoT in healthcare, in which things (in this case sensors) can communicate and analyze data [17], the collection of basic health-related data can be partly or wholly automated, reducing the strain placed on doctors for continuous profiling of health examination data. Furthermore, IoT processes and stores data on a distributed platform [18]. The usage of this platform allows for the development and deployment of machine learning (ML) techniques [19,20] to anticipate the problems with patient health status and to manage hospital/medical resources by projecting future patient needs [4]. Figure 1 presents a practical overview of an IoT-ML-based healthcare delivery system.

The synergy among smart wearable sensors, IoT, AI, and Blockchain technologies in HMS is investigated and presented in this research work. Findings from this investigation will further promote and support the application/adoption of IT in predictive and personalized HMS. Existing health-based concepts such as electronic health (eHealth) [22], mobile health (mHealth) [23], or ubiquitous health (uHealth) [24] have reported successful automation healthcare systems. Primarily, these concepts (mHealth, eHealth and uHealth) successfully utilized ubiquitous and mobile computing technologies [10] to monitor patient health at any time and from any location [25]. Moreover, there is a paradigm shift or a conceptual transition from a reactive to a predictive and personalized HMS [23,24].

Hence, this research work investigates and analyzes existing literature on using smart wearable sensors, IoT, AI, and Blockchain technologies in HMS. Specifically, a thorough review of the application of these emerging technologies (sensors, IoT, AI, and Blockchain) singly and collectively in HMS is explored. The primary contributions of this research work are aptly stated as follows:

- A unique taxonomy that highlights the application of wearable sensors, IoT, AI and Blockchain, in HMS is presented. This taxonomy showcases the strategic steps of the Sensor-IoT-AI-Blockchain-based healthcare system.
- A broad assessment of the deployment of wearable sensors, IoT frameworks, diverse AI techniques and the application of Blockchain technology in the HMS is presented.
- Open research issues that affect the application of these emerging technologies (Sensor-IoT-AI-Blockchain) in HMS are identified.

**Figure 2:**
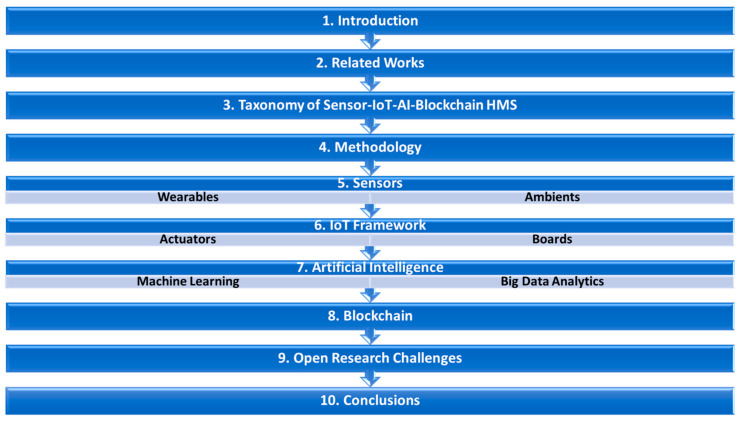
Outline of this study.

This paper is divided into ten sections as illustrated in Figure 2. Section 2 discusses the state-of-the-art related works on HMS. Section 3 discusses the taxonomy of this study based on the Sensor-IoT-AI-Blockchain-based healthcare system. Similarly, Section 4 presents the methodology while Section 5 focuses on wearable sensors and their application in HMS. Section 6, Section 7 and Section 8 detailed the use of IoT, AI and Blockchain technology for HMS with their respective enablers and barriers. Section 9 highlights open research challenges and present future research directions. Lastly, detailed conclusions from the investigation are presented in Section 10.

## 2. Related Works

In this section, associated surveys, and literature on the use of sensors, IoT, AI, and Blockchain in HMS are presented and discussed. This assessment is critical to highlight the inadequacy of thorough evaluations and to indicate the merits and shortcomings of applicable methodologies taxonomically and logically.

### 2.1. Related Studies on the Application of Sensors, IoT, AI, and Blockchain Technologies in HMS

Kamruzzaman, et al. [26] reviewed the application of Internet-of-Medical-Things (IoMT), AI and Edge computing for healthcare delivery in ubiquitous environments. They posited that the aforementioned technologies could assist in the proper monitoring and management of healthcare systems. It was reported that due to the escalation of population and diseases, it is worryingly strenuous for medical staff to adequately attend to patients with relative medical issues. Also, they posited that the introduction of emerging technologies such as AI techniques can solve these rising issues. Yang, et al. [27] reviewed smart HMS and different kinds of sensor components utilized within their respective IoT framework. They categorized and analyzed existing applicable studies based on their leverage of device-based techniques and device-free techniques. They concluded that different combinations of these techniques can be creatively applied to complement existing HMS. Karatas, et al. [28] investigated the application and problems of big data analytics (BDA) as an instance of AI technique. Their review showed that BDA constitutes a vital place in the technology provided in HMS. Alshamrani [29] investigated the applicability and deployment of health Internet of things (H-IoT) based systems in smart cities. In addition, they further assessed HMS-affiliated technologies to create a standpoint on health monitoring using various wearable sensors.

Krishnamoorthy, et al. [30] addressed current communication paradigms such as wireless networks, emphasizing their importance and applicability with emerging technologies such as AI and Blockchain in advancing HMS. They conducted a comparative analysis of several architectural solutions, taking into account their benefits, drawbacks, and quality-of-service needs. Li, et al. [31] in their study, presented a detailed evaluation of the utilization of AI techniques in HMS. Additionally, the strengths and shortcomings of current methodologies, as well as other research obstacles, were discussed. Sworna, Islam, Shatabda and Islam [21] developed a distinct taxonomy from an IoT-ML context to help researchers in future research directions on HMS. Similarly, Tunc, et al. [32] discussed the recent advances in smart HMS. Specifically, a thorough review of IoT, AI, fog computing, and Blockchain as emerging technologies in smart healthcare is presented. Also, Nahavandi, et al. [33] reviewed current applications of wearable sensors based on AI techniques. Literature on the application of ML approaches for medical analysis is examined. Also, use-cases with the combination of IoT and wearable sensors in HMS were discussed.

Qadri, et al. [34] designed an H-IoT framework for disease detection, disease monitoring and assistive living system. The proposed framework was based on prominent technologies such as Blockchain, AI and software-defined networks (SDN). Al-Dhief, et al. [35] offered a general assessment of current IoT and ML techniques used in HMS and demonstrated a voice pathology monitoring system. Some unresolved difficulties and challenges related to the IoT framework in healthcare were also highlighted. Qayyum, et al. [36] focused on the security and privacy problems of AI technologies in HMS. As a solution, an ML pipeline and a classification of alternative methods that assure the security and robustness of AI-based health systems were suggested. Karthick and Pankajavalli [37] presented an overview of the deployment of Human H-IoT (H2IoT) application areas; examined sensing devices and data transmission methods utilized in H2IoT; and discussed problems, privacy, security, and threats in H2IoT. Santos, et al. [38] examined research on online monitoring, detection, and assistance for cardiovascular disease diagnosis. Furthermore, they investigated how to handle security concerns and suggested a standard to assist in developing a cardiovascular monitoring system. Amin and Hossain [39] explored H-IoT applications implemented in edge computing systems. Their research aims to assess current and developing edge computing concepts and approaches for ubiquitous healthcare, as well as to identify the expectations and problems of various application scenarios. Their study also provides a thorough examination of the state-of-the-art AI-based approaches used for edge computing. A similar review was conducted by Alshehri and Muhammad [40] in which they conducted an extensive review on IoT and IoMT-based edge computing HMS.

Dhanvijay and Patil [41] examined wireless communication-based health applications with attention to the network’s configuration, infrastructure, and deployments in healthcare. They examined privacy and security problems in their work and identified certain research needs. Habibzadeh, et al. [42] reviewed the applicability of HIoT in healthcare from the clinical point of view. They depicted HIoT from the viewpoint of three of its key components which are sensing, data transmission and deduction. Mutlag, et al. [43] offered a thorough analysis of the fog computing paradigm in IoT-based healthcare systems. Also, Ray, et al. [44] explored the relevance of edge computing in IoT-based healthcare systems and illustrated several edge-IoT-based healthcare framework use cases. They introduced a revolutionary edge-IoT-based framework for healthcare in their research work. Dang, et al. [45] examined the current trends of IoT-based healthcare systems, as well as emerging technologies such as BDA and cloud computing in healthcare applications, were explored. Furthermore, a detailed assessment of IoT privacy and security problems was performed.

Cui, et al. [46] provided an overview of the use of AI (ML techniques) in IoT. They presented a detailed overview emphasizing current advances in ML for IoT and outline several IoT applications. They posited that the deployment of ML techniques in IoT allows deep insights and construction-efficient intelligent IoT applications. Alam, et al. [47] went on to investigate the major application-based needs derived from the insights of communication-based concepts in HIoT. In addition, many scenarios were utilized to illustrate certain upcoming technologies and standards employed in this industry. In addition, certain predicted trends and difficulties for the prospect of HIoT were identified. Sharma and Singh [48] focused on the deployment of IoT and AI (ML) in diabetes detection and prediction. They presented the applicable concepts and research approaches to diagnosing diabetes and proposed viable frameworks for its detection and prediction. Also, Babu and Shantharajah [49] investigated the use of AI (ML and BDA) and IoT platforms in healthcare. They extensively discussed the advances of ML and BDA in driving actionable insights from health data generated by IoT health-based platforms. In the end, they proposed a unique BDA framework for health data. Sughasiny and Rajeshwari [50] provided a thorough analysis of the significance of AI (ML and BDA) in the health sector.

Farahani, et al. [51] addressed the migration and advancement of HIoT from clinic-based healthcare to HMS. This was achieved with the deployment of multiple wearable sensors and fog computing. The suggested approach transformed the conventional HMS into a more intelligent (smart) HMS. In a similar study, Darwish, et al. [52] developed a cloud IoT-health paradigm by combining cloud computing with the IoT paradigm. Sethi and Sarangi [53] presented an overview of advanced methodologies, technologies, protocols, and applications in IoT infrastructure; examined computing techniques; and emphasized communication technologies in detail. Qi, et al. [54] conducted a comprehensive study of IoT-enabled HMS. They highlighted essential enabling technologies for IoT and contemporary healthcare applications, as well as research obstacles. Tokognon, et al. [55] developed a framework for an HMS system utilizing IoT and BDA elements; they also detailed several communication technologies and protocols for monitoring HMS.

Yuehong, et al. [56] reviewed the deployment of IoT in healthcare as well as described the intelligentization trajectory and continuous research prospects in healthcare delivery. In other words, the implementation techniques and approaches spanning knowledge and resource management are analyzed. Capraro [57] investigated the use of sensors, IoT and AI in healthcare delivery. The study focused on how AI (in this case BDA) can be used to enhance healthcare while minimizing healthcare costs. It was indicated that the successful integration of sensors, IoT, and AI will not only reduce the costs of medical care but also enhance and promote the availability of data for actionable insights for effective healthcare delivery. Similarly, Azzawi, et al. [58] examined IoT and AI in healthcare delivery with attention to its security mechanism. Specifically, a new authentication platform for IoT technologies using Elliptic Curve Cryptography (ECC) method was suggested. In another study, Sakr and Elgammal [59] explored the use of sensors, IoT, data storage mechanisms and AI for quality healthcare services. They developed a data-driven framework, SMART HEALTH, for health analytics and its functionalities in healthcare delivery. Also, Hossain and Muhammad [60] combined sensors, and cloud data infrastructure with IoT technologies for medical services. The proposed H-IoT framework was mainly for monitoring continuous healthcare delivery by using ECG and sensors data for seamless access by healthcare practitioners. Romero, et al. [61] suggested an IoT approach based on the integration of AI and sensors for Parkinson’s disease diagnosis and monitoring. They stated that the growing technicality and acceptance of IoT and wearable sensors in healthcare delivery appears to facilitate an intelligent means of providing ubiquitous medical services to a large population and elevating the technical status of conventional healthcare systems. In addition, Mathew and Pillai [62] discussed the applicability and issues faced by AI (BDA) for deriving insights from health data. Yeole and Kalbande [63] highlighted the many IoT enabling technologies and practices in healthcare delivery particularly for infants, adolescents, terminal diseases, acute clinic services, operating rooms, and prescription dispensers. Their study is based on the premise of enhancing the effectiveness of healthcare delivery systems while minimizing healthcare expenses. Dimitrievski, et al. [64] investigated and gave an overview of the Ambient Assisted Living systems (AALs). Their survey is primarily based on the use and processing of ambient sensor data from IoT devices for activity recognition or classification in HMS.

Islam, et al. [65] explored advancements in IoT-based healthcare technology and examines cutting-edge approaches to IoT-based HMS. Specifically, several IoT security and privacy elements, such as security criteria, threat models, and attack taxonomies were analyzed. Furthermore, an intelligent collaborative security model to reduce security risk was proposed and insights on how emerging technologies such as big data, ambient intelligence, and wearables can be leveraged in a health care context with some avenues for future research on IoT-based health causation. A similar study was posited by [66]. Li, Lu and McDonald-Maier [66] presented a one-stop perspective and overview of the background of Ambient Assisted Living (AAL) research, particularly technology and methodologies for cognitive aided ageing in the home setting. They highlighted successful case studies and solutions that have been implemented. Yang, et al. [67] in a broader perspective examined state-of-the-art information technology (IT) solutions for improved healthcare and suggested workable insights on how IT advances affect healthcare practices. Wahaishi, et al. [68] proposed an agent-based architecture that permits ad-hoc system setups, highlighting tactics for achieving real-time smart monitoring in SmartHealth settings with a privacy-based communication mechanism to preserve individuals’ identity while exchanging sensory information.

### 2.2. Comparison of Existing Literature with This Study and Motivation

**Figure 3:**
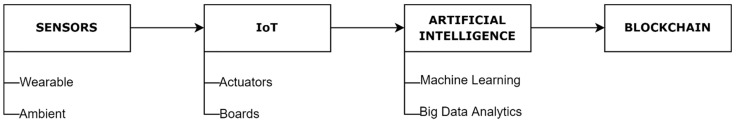
Taxonomy of Sensor-IoT-AI-Blockchain-based Healthcare.

In this section, this survey is compared with the existing surveys and literature as depicted in Table 1. Part of the contribution of this survey is that it examines the identified concepts highlighted in Table 1 using different cases. Several studies have reported these concepts singly or in pairs [15,26,31,32,33,34,40,43,44,52]. Nonetheless, this survey combined and examined all these concepts as a distinct taxonomy (See Figure 3). In other words, the primary distinction between this study and the other existing literature as shown in Table 1 is that we developed a new taxonomy with the notion of Sensor-IoT-AI-Blockchain-healthcare. As illustrated in the taxonomy, the highlighted concepts and subareas are discussed in depth throughout this study.

Another novel addition is that, unlike previous survey papers, we gathered research publications in line with the suggested classification and its sub-classifications. We then organized the whole survey papers around that, demonstrating different kinds of wearable and ambient sensors with their application in HMS. A thorough analysis was conducted to discover several kinds of sensors utilized in HMS for a range of ailments and observed findings are summarized and presented. In addition, actuators and development boards are discussed as part of IoT infrastructure. Another important feature of this study is the distinctive explanation of the application of AI and Blockchain technologies with the Sensor-IoT-AI-Blockchain-based HMS. To the best of our knowledge, such a presentation and categorization of the AI and Blockchain sections are novel in comparison to existing survey papers [36]. All these significant enhancements make this study detailed and balanced when compared to other surveys.

## 3. Taxonomy

In this section, we propose a taxonomy for research in Sensor-IoT-AI-Blockchain-based HMS, identifying four critical concepts/layers as depicted in Figure 1. The taxonomy’s primary objective is to investigate the deployment of Sensor-IoT-AI-Blockchain in HMS.

The starting point of the taxonomy is the sensors. Sensors are devices that generate output signals to detect physical phenomena. In other words, sensors are used for data collection in an environment or domain of interest. An instance of such a domain is the healthcare sector where sensors are used to gather health-related data (actively or passively) from a patient. These data are either transmitted to another sensor or analyzed. An example of sensors used in healthcare is the medical sensor. Medical sensors are specially designed and deployed to measure and monitor a patient’s vital signs such as heart rate (HR), oximeter, pressure, glucose, temperature, Electrocardiogram (ECG), Electroencephalogram (EEG) and so on.

The next component is the IoT architecture. IoT is a web of smart and self-configuring things (devices) that can communicate with each other using a communication link or network (in this case the Internet). It is essentially a cyber-physical system or a network of networks consisting of different kinds of devices (sensors). It is worth noting that sensors can be discussed as a component of the IoT architecture since it is primarily used for data collection from an environment. However, the IoT architecture is beyond the communication of connected devices. Actuators, development boards, communication and storage infrastructure are components of IoT architecture. Actuators are employed to impact continuous alterations in the surrounding. The temperature controller of an air conditioner is a typical example of an actuator. Also, development boards are used to design the system to collect, process, and transport data to various sorts of systems. Arduino, Raspberry Pi, Intel Edison, and so on, are examples of the development board.

The third component is AI. The concept of AI as used in the healthcare domain is presented in Section 7. In this survey, AI is broadly divided into core ML and BDA. The essence of this categorization is to generate a broader conceptual view of the applicability of AI in HMS. Details on ML and its characteristics may not be sufficient to portray the adequacies of AI in healthcare. Hence, the category of BDA is added to show how the humongous data generated from IoT-based HMS are being analyzed and converted into actionable insights. Different sub-categories of ML such as Supervised, Unsupervised, Semi-Supervised, and Reinforcement ML techniques in healthcare are investigated. Also, BDA technologies concerning HMS are discussed in detail.

At the same level as AI is Blockchain technology (Fourth component). It is somewhat of a new concept that has not been wholly and extensively utilized in HMS. Blockchain is a shared, immutable ledger that facilitates the process of recording transactions and tracking assets in a business network. In other words, Blockchain technology is a decentralized, distributed, and public digital ledger that is utilized for saving the transaction in various nodes. Therefore, any third person involved cannot alter records because every block has a cryptographic value of the previous block and itself. One of the prominent applications of Blockchain in HMS is in the creation of a shared archive of health-related data for doctors and patients independent of their electronic diagnosis, improving safety and privacy, investing fewer resources in the medical staff and more money in patient care.

Details on the applications of each of the components in HMS have been added to their respective sections to aid readability and ease of understanding of this study.

## 4. Methodology

This survey employed the PRISMA method for the selection of research papers to be included in this study.

### 4.1. Inclusion and Exclusion Criteria

This research systematically attempts to explain the necessity for and use of emerging technologies (Sensor-IoT-AI-Blockchain) in the present healthcare domain, offering a detailed assessment of these technologies as well as the prior techniques and methodologies engaged in the current scenario to assist the system [69,70].

Particularly, this survey contains publications and survey papers that are directly linked to sensors, IoT, AI and Blockchain deployment in HMS. This gives insights into hundreds of the publications included in this study, as well as the methodology used in existing studies.

In this study, the paper selection criteria procedure is separated into three subsections: keyword selection, inclusion and exclusion, and the final findings generated utilizing these approaches. Details on these selection criteria are presented in the following sub-sections.

#### 4.1.1. Selection of Keywords

A comprehensive search for research articles was undertaken in various well-known databases, including IEEE, Science Direct, PubMed, Wiley, Taylor and Francis and others. Keywords such as sensors, wearable sensors, biosensors, IoT, AI, ML, Blockchain, healthcare, telemedicine, and e-health were used to search the articles in the aforementioned databases.

#### 4.1.2. Inclusion

The research included only articles published from 2015 to 2022, with the remainder being eliminated. These selected articles were chosen for review after being reviewed with the abstract as the focal point and papers particularly stating the use of wearable technology, IoT, AI, and Blockchain and its relevance to this study. This research contains the analysis of research articles, current review papers, technical notes, and other materials grouped in a systematic sequence linked to recent advances in wearable sensors, IoT, AI, Blockchain and HMS.

#### 4.1.3. Exclusion

During the search for research articles, there are some strong criteria for exclusion of papers which includes duplication of papers, language (English language only), and irrelevant papers (subject and material). Papers were also removed if they had no connection to wearable technology and offered previously published material on the same subject. Also, case series and reports, short communications, editorial notes, and other materials were excluded.

### 4.2. Quality Assessment and Data Extraction

It is vital to remember that the number of articles, including surveys relevant to the healthcare domain, has been expanding, with many academics attempting to add to the literature. However, such studies (reviews or surveys) are prone to some flaws, including, increasingly, nonrandomized intervention studies. Authors must be able to identify high-quality reviews or surveys. Many ways for analyzing individual aspects of feedback have been devised, but there are few systematic instruments for critical assessment [71].

**Figure 4:**
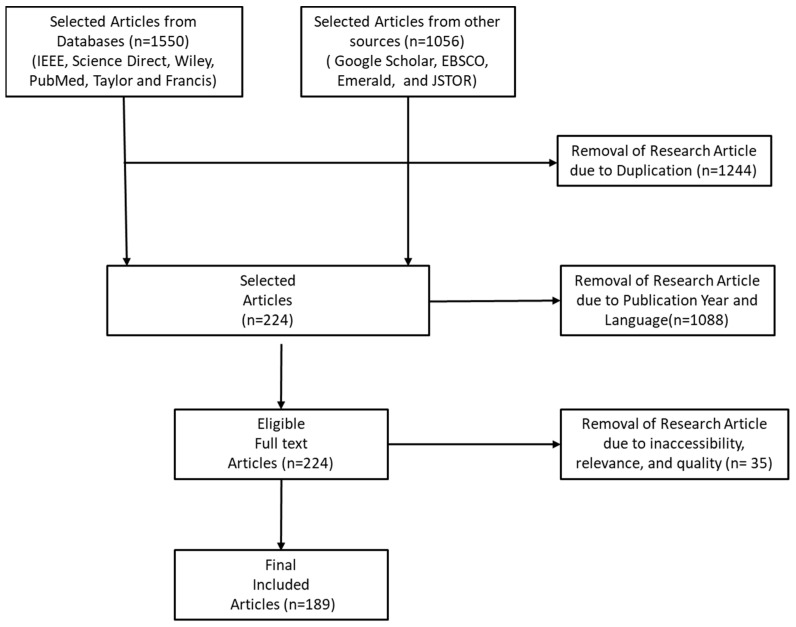
PRISMA Process for selecting relevant articles.

In this respect, the PRISMA technique is used in this research to evaluate the quality of the chosen articles and guarantee that the articles included in the review are of high quality. Furthermore, the PRISMA technique is used to critically examine the material important to each of the chosen papers [71]. Publications were selected utilizing the inclusion and exclusion criteria, including the year of publication (as established by the PRISMA checklist). Figure 4 depicts the PRISMA method for this survey.

Consequently, following the thorough assessment of 2606 articles gathered from multiple sources (IEEE, Science Direct, Wiley, Google Scholar, EBSCO, PubMed, Taylor and Francis, Emerald and JSTOR) in the first stage, 1244 articles were removed due to duplication across the selected repositories. A further 1088 articles were excluded based on the considered publication year of the articles and 35 articles were removed due to inaccessibility, relevance, and quality of some of the articles. In the end, 189 articles are selected after full review and assessments.

## 5. Sensors

Sensing is a technology that is employed in practically every element of hospital-based care, from the most basic device such as a thermometer to complicated precision-based equipment. For example, digital image sensor technologies are regularly employed technology to give medical professionals with many insights into each patient’s health status [72]. These sensors have been significant in revolutionizing diagnostic medicine. Such information, in particular, enables doctors to detect regions of damage or abnormalities, perform microsurgical procedures, and analyze the outcome of a procedure [73]. In obstetric treatment, for example, ultrasonography allows the physician to follow and analyze the growing foetus and identify any foetal or other anomalies that may damage the mother’s or baby’s health [74]. Pathologists also use modern sensing technologies in hospital labs to conduct haematology, immunology, biochemistry, histopathology, and microbiology activities. Sensors may also play an important part in medical therapy. They can, for example, identify events such as missing heartbeats. Furthermore, they can be utilized in improving the medication administration procedure by selecting the best moment to administer medicine [6,75].

Examples of significant sensor application areas in HMS include [41]:

- Medical Imaging: The charge-coupled device (CCD) and other applicable sensor devices are widely used for medical imaging [45]. These devices are used to gather still or moving images inside the patient. A prime application is the Smart Pills for gastrointestinal imaging [40]
- Screening and Diagnostics: Ocular and metabolic sensors are employed in diagnostics and bedside testing [72]. Besides, metabolic sensors can be used to detect medicines, protein expression, viral infection, and endocrine systems in biological fluids or specimens [42].
- Movement and Trajectories: Wearable sensors such as accelerometers and gyroscopes can be used to assess balance and fall risk, as well as monitor the effect of medical therapies. Motion sensors are used to evaluate prosthetic limb replacements. They are also used in stroke therapy to monitor the progress of certain physical activities.
- Physiological: With the aid of an applicable sensor, vital health signs related to the physiology of a patient such as HR and BP can be monitored [39].

In general, sensors can be categorized into wearable and ambient (environmental sensors).

### 5.1. Wearable Sensors

Wearable sensors are beginning to appear, offering patients with cheap and uninterrupted evaluation of medical symptoms independent of place and health status. It is expected that 5 million of these sensors would be marketed by 2018 [6]. Wearable sensors, which are becoming more convenient and unobtrusive, are critical in assessing a patient’s health or well-being without interfering with their everyday routines. By inserting the sensors in various areas of the body, the sensors can monitor a range of vital signs likewise a patient’s behaviour and mobility. Ultimately, minimal power, condense and cheap devices and current IT infrastructure are paving the way for cheap, discreet, and lifelong wearable sensors. The LifeShirt, for example, is a multi-sensor extended HMS for gathering and analyzing the health data of a patient [76]. It captures continuous patient data instead of data fragments, that are generally obtained through periodic doctor’s physical examinations. Table 2 presents a further categorization of wearable sensors into inertial sensors, location sensors, physiological sensors, and image sensors with applicable examples.

#### Literature on Wearable Sensors in Healthcare

This sub-section presents and analyses research articles on the deployment of wearable sensors in healthcare. Studies such as [44,77,78,79,80,81,82,83,84,85,86] addressed the deployment of wearable sensors in healthcare.

Ray, Dash and De [86] proposed a portable and low-cost galvanic skin response (GSR) device that amplifies, captures and processes GSR data in smart e-healthcare applications. The GSR device evaluates a patient’s degree of behaviour and movements which is analyzed and presented on the patient’s devices. Bhatia and Sood [79] developed a smart HMS for real-time medical analysis of its users based on an ML approach. The suggested HMS works by aggregating data from its user via smart wristbands for the prediction of health status risks using an artificial neural network (ANN). The results of the experiments reveal that the proposed HMS is effective in analyzing patient’s health status. Azimi, Pahikkala, Rahmani, Niela-Vilén, Axelin and Liljeberg [78] designed a customized robust decision-making approach for providing medical options. They experimented on pregnant women who wore wristbands to monitor their maternal health and proved that their approach is effective in monitoring maternal health state. Also, Yang, Zhou, Lei, Zheng and Xiang [77] suggested a compact wearable gadget for long-term ECG signal detection with low cost and high performance. Similarly, Wu, Wu, Redoute and Yuce [80] designed a wearable sensor-based system to detect falls by analyzing the change in body temperature and heart rate. Niitsu, Kobayashi, Nishio, Hayashi, Ikeda, Ando, Ogawa, Kai, Nishizawa and Nakazato [83] proposed an environmentally friendly and high-performance self-powered disposable supply-sensing biosensor platform employing an organic biofuel cell for big data-based healthcare applications in IoT. Hallfors, Alhawari, Abi Jaoude, Kifle, Saleh, Liao, Ismail and Isakovic [85] described the characterization, manufacture, and effectiveness of wearable IoT-based sensors constructed from graphene oxide (rGOx). It was observed that the rGOx performed well in terms of noise level for ECG signal amplitudes. Furthermore, the experimental findings demonstrated the recommended architecture’s great sensitivity and predictability. Esmaeili, et al. [87] suggested an effective lightweight device that classifies patients’ health data based on a priority algorithm and provides emergency assistance with little delay. Muthu, et al. [88] created an IoT-connected wearable sensor with ML to forecast illnesses, notify patients, and deliver therapies. Huifeng, et al. [89] developed IoT-based wearable sensors with an integrated ML support that continuously gathers health metrics and track activity to evaluate sports performance and the health of the patient. Wu, Wu, Qiu, Redouté and Yuce [82] created a small wearable sensor patch that measures vital signs such as body temperature and allows remote monitoring of a patient’s health status. However, regardless of the successful deployments of these wearable devices as reported in these studies, security and privacy problems are still a problem.

### 5.2. Ambient Sensors

Ambient or environmental sensors are utilized to detect and measure physical environmental changes. Air pollution is responsible for the worsening of various physical health conditions including asthma and lung cancer [90]. Temperature, humidity, and air pollution can all be measured using these ambient sensors. Table 3 illustrates an extended categorization of ambient sensors into further divisions and tags with examples.

#### Literature on Ambient Sensors in Healthcare

This sub-section presents and analyses research articles on the application of ambient sensors in healthcare.

Vilela, et al. [91] developed a fog-assisted health monitoring system for real-time applications and demonstrated its excellent performance and security in a hospital setting. However, interoperability across heterogeneous devices was a significant problem in their study. Ray, et al. [92] developed a non-invasive, low-power, and cost-effective sensor system for monitoring intravenous (IV) fluid bag levels in real-time in e-healthcare applications. Caregivers may use this application to check the status of the IV fluid bag on the web page to determine whether it was about to run out. Furthermore, Elsts, et al. [93] proposed a multi-modal platform called SPHERE and employed it in smart home applications to cut power consumption and costs associated with monitoring people in residential settings. SPHERE’s network design accommodated both software and hardware needs, according to the authors. Also, Chen, et al. [94] suggested three techniques to optimize packet size and power management choices in e-health WSNs. The performance, latency, dependability, and longevity of these three methods were compared.

### 5.3. Commonly Deployed Sensors in Healthcare

The most significant components of the wider HMS are the health sensors. They are used to detect various health issues and to record health data. Health sensors are sometimes referred to as biosensors since they are placed on or inside the body to read vital signs (health data). Under the auspices of an IoT-enabled ubiquitous healthcare environment, many kinds of health sensors are developed to detect different kinds of vital signs in the patient body. The operation of these gadgets may differ depending on their use. Hence, a variety of biosensors that assist ubiquitous healthcare systems are covered in the following subsections.

#### 5.3.1. Blood Pressure (BP) Sensor

The purpose of measuring blood pressure (BP) is to quantify the force that travels through the blood channels against the artery wall. Typically, a sphygmomanometer is used for measuring BP. If the flow of blood in the artery is normal, the BP is normal. If blood flow is impeded by any cause, BP rises. High BP may lead to serious health complications [95]. The BP measuring procedure finds two pressure points within the blood channels: one when the heart is beating (systolic) and another whilst the heart is at rest between two heartbeats (diastolic). Today, various standard BP testing techniques are accessible. The “gold standard” is a mercury sphygmomanometer. The digital manometer measures BP using an electronic pressure sensor and is based on the oscillometric concept; the values are shown digitally on a display [96]. This technique of measuring BP is simple and may be linked to a smart hub, such as a smartphone, to support a comprehensive healthcare system.

#### 5.3.2. Body Temperature (BT) Sensor

Body temperature (BT) is one of the most crucial indicators of a patient’s physiological status and the average human BT is about 37 °C. A high BT may be a common symptom of most illnesses or insufficient blood flow owing to circulatory shock [97]. A healthy person’s BT value may also change somewhat depending on the time of day and the location of the measurement on the body. As a result, while measuring the BT, caution must be taken to properly calibrate the temperature measurements. Wearable BT sensors are often utilized in the ubiquitous healthcare setting and are put on the body. Sensors placed on the body have a temperature that is roughly 5 °C cooler than the body’s core temperature. Due to their higher sensitivity and accuracy, thermistor-based sensors are chosen for BT monitoring in wearable and other non-invasive technologies [95]. The resistance of a thermistor varies with temperature changes. The resistance of one form of a thermistor, known as a positive temperature coefficient (PTC) type, increases as the temperature rises. The resistance decreases with decreasing temperature in the other category, which is known as a negative temperature coefficient (NTC) type thermistor. The BT sensors can be deployed in different ways depending on the mode of operation. For instance, the BT sensor can be attached to clothes like in the case of wearable fabric for babies that uses conductive textile wires for its BT sensor [98]. Also, LM35 is another wearable BT sensor that is based on integrated circuits (ICs). The BT sensors can be placed on the patient’s skin for periodic temperature assessment.

#### 5.3.3. Electrocardiography (ECG) Sensor

Electrocardiography (ECG) is one of the oldest and simplest diagnostics for determining important information about a patient’s cardiovascular system [95]. An ECG depicts the electrical impulses of the cardiac muscles in reaction to electrical discharge on graph paper. Various procedures may be used to perform an ECG. Wet ECG, in which electrodes are placed on the chest, arms, hands, and legs, is a traditional technique that employs a unique kind of conductive gel that acts as a conducting medium for electrical impulses from the body to the electrodes. Long-term usage of wet ECG has downsides such as patient allergies owing to contact with metal electrodes and gel, or surface degradation of electrodes leading to a decrease in signal quality [99]. Regular assessment of the ECG signals can assist in identifying abnormal signs and arrhythmias in high-risk individuals [96,100]. In such cases, an alteration from primary ECG readings can be detected, and adequate medical care can be given to such a patient. In today’s ubiquitous HMS, such a system is achievable in which patients may go about their everyday routines while being continually assessed for ECG signals. Another approach to measure ECG is to deploy the Capacitively Coupled ECG. CC-ECG is a way of obtaining an ECG signal without physical contact with the patient [95]. In this procedure, a tiny layer of insulator separates the human body from a metal plate electrode, resulting in the formation of a capacitor. The electrodes may be attached to a fabric that is worn by the individual who requires continuous monitoring of ECG readings. To enable a ubiquitous healthcare environment, the CC-ECG sensor-based ECG may be built to be portable and compact, as well as wireless. To extend battery life, low-power components such as idle mode, low-power wireless protocol, and so on are used.

#### 5.3.4. Electroencephalogram (EEG) Sensor

The electroencephalogram (EEG) is a method for measuring the electrical impulses of a person’s brain using tiny electrodes placed at various sites on the scalp [101]. It is a non-invasive procedure that may be used on patients, healthy adults, and children repeatedly with no dangers. Nerves activating in the brain produce electrical impulses with amplitudes in the microvolt (mV) range and frequencies ranging from 8 Hz to 50 Hz. EEG is employed in a wide range of medical and non-medical scenarios. Assessing brain activity; detecting regions of interest after a head laceration, seizure, tumour, and so on; researching sleep disorders and physiology [102], and so on are some of the medical uses. Among nonmedical uses, EEG is utilized for the psychological training of athletes, assisting them in improving attention and effectively managing stress or weariness. EEG may also be utilized to investigate cognitive processes, decision-making, driver awareness, and so on [103].

#### 5.3.5. Pulse Oximeter

The amount of oxygen in the blood is an essential characteristic of the human body. The human body may struggle to function correctly if the oxygen level is insufficient, and very low oxygen levels may strain the heart and brain. A blood-oxygen saturation level reveals the proportion of haemoglobin molecules in arterial blood that is saturated with oxygen in comparison to their maximal possible absorption value. Under typical circumstances, the oxygen saturation level is more than 89%. A pulse oximeter is a device for measuring the blood-oxygen saturation level [104]. The pulse oximeter calculates the proportion of haemoglobin bound to oxygen based on the quantity of red and infrared light absorbed. Sensors may be put on the earlobe or the toe in addition to the fingertip. In the ubiquitous monitoring environment, the sensor unit’s signals interface with a miniature processing unit, which then transmits the data to the healthcare unit through wireless methods such as WLAN, and so on [105].

#### 5.3.6. Heart Rate (HR) Monitor

The human heart oversees pumping oxygenated blood and nutrients to various regions of the body as well as collecting deoxygenated blood that contains carbon dioxide. A cardiac cycle is defined as the stages involved in converting deoxygenated blood to oxygenated blood in the lungs and pumping it via the aorta by the heart [106]. The frequency of the cardiac cycle known as the heart rate (HR) is measured in beats per minute (BPM). HR is a critical indicator to determine a patient’s mental and physical wellness. HR for an average healthy adult varies between 60 BPM and 100 BPM. A low HR value typically indicates improved cardiovascular health, heart fitness and effective heart function. A patient with poor physical fitness typically has a high HR value. HR can be measured using a variety of ways one of the least is the intrusive and near-infrared light often used in popular procedures [107]. The sensor configuration is like the pulse oximeter described in the preceding section and operates on the same principles. Wrist wearable HR monitors that can connect to the internet through mobile phones for data storage and further data analysis by doctors are now widely available and accessible for promoting prompt healthcare delivery [106].

#### 5.3.7. Motion and Activity Sensor

Physical exercise is a key factor in maintaining a healthy body and physique. Furthermore, a patient’s mobility is a crucial component that must be closely watched throughout the rehabilitation time in a hospital. A pedometer, actometer, or accelerometer are the most common sensors used to assess a patient’s movements and physical activity. A pedometer detects the action of hands-on-hips to tally each step. A GPS receiver may also be used to calculate the distance travelled by a person. Actometers may measure acceleration and intensity of movement. A recent study indicates that identifying diverse body positions such as sitting, lying, and walking [108] is also achievable to provide a novel ambulatory assessment method, particularly for senior (aged) patients. To detect such positions, little kinematic sensors are implanted on the person’s chest. Furthermore, the sensors have aided in the identification of the postural transition between standing, sitting, lying, and locomotion activities when standing.

### 5.4. Challenges and Open Issues of Sensors in Healthcare

Wearable sensors are often accessible nowadays in the form of smartwatches that may link to smartphones. Wearables are projected to appear in a variety of forms intended for specific uses in the future. In the future, it is expected that wearable sensors can assist humans in carrying out their responsibilities. The present crop of wearable technologies is far from flawless. Although the sensing technologies are amazing, they are not yet mature enough. Multiple hurdles must be overcome before the full potential of wearable sensing technology may be realized. Some of the issues that wearable sensing technologies encounter are covered briefly in the following subsections.

#### 5.4.1. Data Collection

A notable difficulty of wearable sensors is data collection. The wearable device determines the quality, quantity, resolution, and other aspects of the collected data. Factors such as spatial resolution, temporal resolution, and data resolution may all influence data quality and quantity [72]. It is difficult to collect data from sensor users in an efficient way. Before it can be used in clinical trials, raw data must be pre-processed. To that aim, the measured values from various devices must be harmonized, as well as their error and statistical outliers eliminated. The data are ready to be utilized by data analytics once they have been pre-processed. ML, a form of AI, is often used in data processing solutions for wearable data [109]. Obtaining high-quality labelled data from sensors takes time and it usually involves specialist knowledge or the assistance of the wearable user in categorizing such data [110].

#### 5.4.2. Data Transmission

It is critical to provide an energy-efficient approach for transmitting data (collected by sensors) for further processing. The introduction of faster connectivity technologies, such as 5G and beyond are beneficial for prompt data transmission, however, these connectivity technologies could lead to an increase in data creation which will require proper and adequate planning for processing and storage. Due to data processing delay and high pressure on network performance, relying only on a centralized data storage system is not a solution. Distributed computing may minimize latency by relocating processing to the network’s nodes. However, there are still challenges with the development of node device software and hardware that must be addressed to satisfy the distributed computing demand [111].

#### 5.4.3. Security and Privacy

Enforcing privacy, security, and trustworthiness while wearing wearable sensors remains a difficulty [6]. The wearables’ major characteristic is constant sensing and data collecting. As described in Mamdiwar, Shakruwala, Chadha, Srinivasan and Chang [74], most current wearables can gather data on the user’s location, physical activity level, and mental health. From the user’s perspective, this data may be deemed sensitive, therefore protecting their privacy is critical. There is currently no comprehensive solution to handle all the possible security and privacy issues posed by wearable sensors, thus further research and development is necessary to enhance the security and privacy elements of wearable devices.

#### 5.4.4. User Acceptance

The acceptance of wearable technology by target end-users is directly proportional to their success. User adoption is particularly difficult in medical and industrial applications. In all other cases, user adoption is a question of personal preference. Wearables, on the contrary, are more of a need than a choice in the medical and industrial spheres. Patients in the medical field may experience pain and stress because of using diverse medical equipment. This is mostly due to the wearable devices’ complexity and excessive intrusiveness. Some employees in the industrial sector may not grasp the usefulness and purpose of monitoring wearable devices and may oppose utilizing them.

#### 5.4.5. Scalability and Interoperability

The exact localization of wearable devices is critical in many applications of wearable technology. Given that wearables are often resource-constrained, attaining adequate accuracy in localization is difficult. As a result, it is necessary to improve the localization quality of wearable devices while working with limited processing resources. Wearable devices, regardless of technology, must be able to interact with one another. This kind of device-to-device (D2D) communication between wearable devices with varying computing capabilities (e.g., low-end, and high-end devices) is a means toward the decentralized implementation of many smart systems. Recall that a single wearable gadget does not have much to give because of its scant resources. However, with proper management and D2D connectivity, as well as the combined computing power of numerous wearables, they may be combined to complete difficult jobs. Furthermore, to fully benefit from the IoT, end-to-end solutions for seamless integration of wearable objects must be developed. One of the major issues is integrating new systems with current ones.

#### 5.4.6. Resource Constraints

Providing new services and reaching out to new people necessitates the development of enhanced wearable functions. Adding additional functions, on the other hand, increases the power consumption of already resource-constrained wearables. Due to constrained resources, the quality of the final wearable product may not always be fulfilled. As a result, one of the most fundamental difficulties of wearable sensors is limiting energy consumption while attaining desired performance.

## 6. IoT Framework

This section discusses the second component (That is, IoT) in the taxonomy. At first, a description of an IoT framework for HMS is presented. Thereafter, each section of the IoT framework about HMS is further discussed.

The primary concept in support of deploying IoT and its mechanisms in healthcare is twofold: (1) hesitation from patients to physically see medical doctors; and (2) real-time medical assistance/attention to patients. As a result, ubiquitous healthcare devices are utilized to prompt people’s responses to bodily ailments. There is no one generalized design for IoT that researchers can agree on. IoT frameworks were offered in a variety of ways by various researchers. The most fundamental IoT architecture comprises three layers: sensing, network, and application. It provides the core notion of IoT in a very brief way, which is inadequate for research purposes. As a result, we provide a four-layer design in which the Network Layer is further subdivided into the Communication Layer and the Network Storage Layer.

### 6.1. IoT Layers

The IoT layers can be broadly categorized into 4 layers namely the Sensing Layer, Communication Layer, Network Storage Layer and Application Layer.

**Figure 5:**
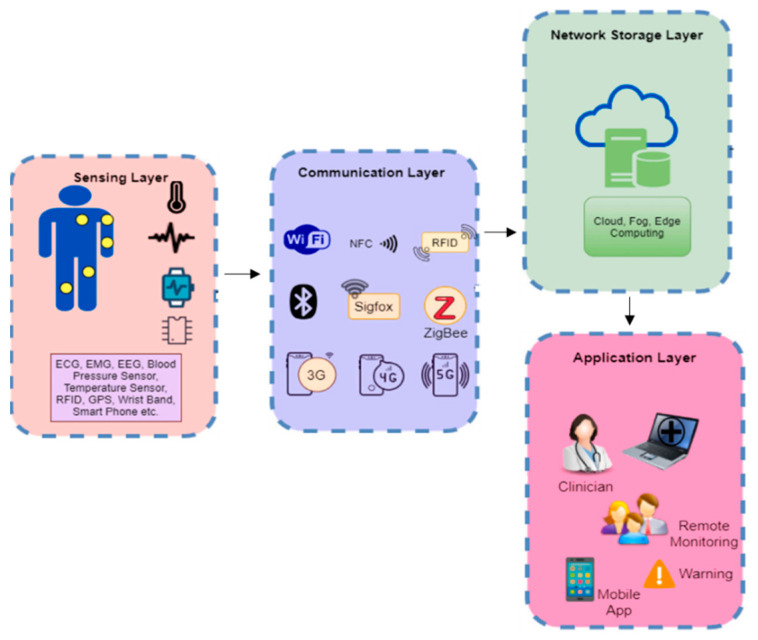
The IoT 4-Layer Architecture for HMS [21].

The sensing layer is positioned in the layer closest to the patient. This layer oversees data collection. It is made up of multiple wearable sensors that patients carry or wear and is intended to monitor a patient’s health status [112]. Figure 5 depicts many sensors integrated to detect physiological data from the human body, such as ECG, EEG, GPS, BT, BP, glucose measurement, and so on. Studies such as Tabassum, et al. [113] and Magaña-Espinoza, et al. [114] successfully utilized smartphones to gather data from the human body. In the Communication layer, the sensor data gathered are transported to the network storage level using different protocols and technologies. Several protocols, including ZigBee, Wi-Fi, Bluetooth, NFC, RFID, and others, are used at this level, depending on the kind of sensor and data. The Network Storage Layer is the third layer of the IoT framework. The advances in IoT technology have increased the number of users, which has increased the amount or size of data. This problem inspired the concept of IoT cloud-based architecture. In general, this layer is in charge of storing and handling vast amounts of data. It also aids in reducing the strain on a sensor device [77]. The Application Layer is primarily about data visualization. It mostly employs application-based services to gather actionable insights from the vast sensor data (health data). It enables easy access to data saved on the IoT cloud server and it aids the doctors, nurses, and patient’s families to track a patient’s health status. If a patient’s health state deteriorates, devices at the application layer can send appropriate messages to the stakeholders. In general, several use cases, such as a smart home monitoring system, a smart city system, a smart transportation system, activity recognition system, disease prediction and so on [21] can serve as the application units in the IoT framework.

Aside from sensors, some other devices such as actuators and development boards are also integral to the IoT framework. Therefore, the following subsections explain actuators and development boards as IoT components.

### 6.2. Actuators

An actuator is a device that can affect the surroundings. It transforms electrical energy into a different kind of energy. To put it another way, an actuator works contrary to a sensor. It accepts electrical energy as input and converts it into physical activity. In IoT solutions, actuators come in a variety of forms, such as automated light on/off in a smart home system. A “Home Service Robot,” for example, may be utilized to provide healthcare services [115].

Actuators that cause motion are categorized into three types based on their operation: electrical, hydraulic, and pneumatic actuators. Hydraulic actuators enable mechanical motion by using fluid or hydraulic power. Pneumatic actuators employ compressed air pressure, whereas electrical actuators use electricity. Consider a smart home system, which is made up of several sensors and actuators. These actuators are used to lock and unlock doors, turn on and off lights and other electrical appliances, inform users of potential hazards through alarms or alerts, and manage the temperature of a house (via a thermostat). A complex example of an actuator used in IoT is a digital finger, which is used to turn on/off switches (or anything that needs little motion) and is operated wirelessly.

### 6.3. Development Boards

Development boards are referred to as the heart of IoT and they primarily work following the needs of the system or application. These boards can often be regarded as small gadget that functions similarly to a computer but with one task/process at a time. Development boards are primarily used to receive, monitor, analyze, and record sensor data from patients. These data can be forwarded to the storage/cloud server for additional analysis and processing. Many devices (sensors) may be immediately connected to a development board to create a complicated system that monitors several vital signs. Since numerous kinds of development boards can be utilized in healthcare, any one of these devices can be chosen based on needs or requirements. Table 4 presents the comparison of some popular development boards based on their characteristics.

### 6.4. IoT in Healthcare

**Figure 6:**
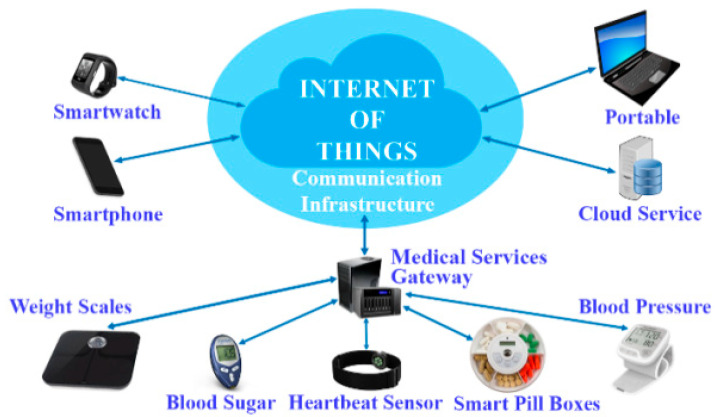
IoT devices in Healthcare [35].

Despite significant investments in IT by the healthcare sector, organizations in healthcare today still rely on conventional (paper) medical processes for doctors and caregivers to communicate with patients. As a result, data exchange between departments and doctors is difficult and restricted, with doctors obtaining information solely via physical examinations during the patients’ hospital visits. However, this problem can be surmounted with the integration of IoT with HMS. Incorporating IoT in healthcare allows doctors and caregivers to access the patient’s information and health status freely readily and easily. Furthermore, public health monitoring, treatment, and diagnostics may be carried out more conveniently and cost-effectively. In other words, IoT can link smart devices, people, and machines to create an effective HMS [35]. Nonetheless, the patient’s continuous health assessment is a critical venture in the healthcare industry. As reported by the US Institute of Medicine, medical mistakes continue to kill three individuals out of every 400,000 people each year. The major cause of these mistakes is failure to perform adequate tests or lack of continuous consultation with physicians, late diagnosis, and inability to obtain a patient’s health profile or history [116]. As a solution, IoT can continuous aggregate health data from various devices and display actionable insights in real-time [117]. Figure 6 depicts several IoT applications in the healthcare industry, where IoT devices may be used for a variety of functions such as vital signs assessments and monitoring.

**Figure 7:**
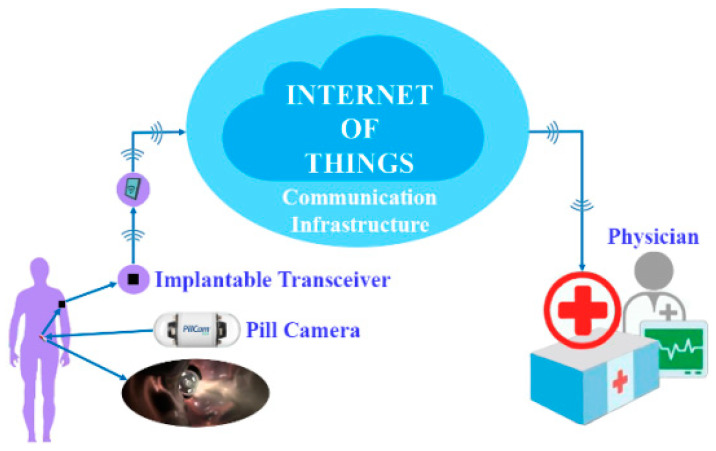
A depiction of a remote healthcare system based on IoT [35].

Consequently, diagnostic findings may be trusted, and appropriate therapy can be provided to the patient. Doctors and caregivers may effectively manage and monitor patient health with IoT technology, saving health response time. There is no need for regular physical visitation of patients when using IoT in healthcare since caretakers or doctors may offer remote health tracking and diagnostics. When receiving the detected information, the proper department may be identified via Wi-Fi and sensors in the hospital [118]. Furthermore, patients in intensive care (clinical or otherwise) can be periodically tracked utilizing IoT mechanisms. For example, the medical sensor takes physiological data from the patient to be studied and then transmits it through gateways. The information gathered will be saved in the cloud. As a result, the patient’s care quality increases while the cost of treatment falls. Meanwhile, an IoT-based remote health monitoring system may follow a patient’s health status in real-time and react if there is an issue with the patient’s health. As seen in Figure 7, a sensor device may be mounted on a patient and the patient’s vital sign data can be transmitted from the same location. The transmitter is linked to the hospital through the communication link or network [119]. The hospital’s technology remotely checks and analyses the patient’s health data.

### 6.5. Challenges and Open Issues of IoT in Healthcare

Numerous studies have contributed to the design and implementation of different IoT-based HMS, as well as the resolution of various technical and architectural issues linked with such systems. Aside from the research problems raised in the literature, there are several additional obstacles and unresolved questions that must be properly addressed. This section briefly highlights both known and unsolved challenges related to IoT healthcare services.

#### 6.5.1. Data Management

Using IoT in medical care, health-related data are gathered from various devices, sensors, and instruments, and sent to a server connected to the Internet that holds databases through gateways. Certain communication standards in the network interface exist between devices and gateways. The interface between gateways and databases will also regulate some of the requirements that require the usage of recognized standards and certifications. The fundamental issue is that many device manufacturers do not comply with these requirements and certifications. This will cause interoperability issues as well as an increase in system costs.

#### 6.5.2. Scalability

Since connected operations are becoming increasingly sophisticated with the inclusion of different applications because of the exponential rise of demands from both people and health organizations, IoT healthcare services, networks, databases, and applications should be scalable. Many devices are interconnected in the IoT system, and these devices generate vast volumes of data that must be absorbed, stored, and processed to draw relevant conclusions. The kind of data generated by each device will vary; for example, medical equipment generates picture data, while other devices generate video data, and so on. This leads to conventional big data challenges, for which infrastructure and platforms are insufficient. The performance requirements for applications and devices will differ, increasing the data-processing mechanisms in the IoT healthcare ecosystem.

#### 6.5.3. Security and Privacy

It is vital to safeguard the aggregated data from various devices and sensors in healthcare against unauthorized access. Unauthorized access to data obtained by medical devices must be avoided. This medical data must only be used for the reasons for which the patient obtained consent. Policies and protocols must be followed to guarantee that only authorized people and applications have access to the patient’s medical data. To exchange health data with authorized people, companies, and applications, strict regulations and technological security measures should be implemented. An open task is to develop an ideal algorithm for cooperation amongst protection, detection, and response services to prevent different attacks, threats, and vulnerabilities.

#### 6.5.4. Interoperability

Interoperability is a critical aspect of HIoT resources and data exchange between patients and sensing devices. The main challenge of interoperability is developing open-source frameworks with a consistent connection; a set of standards must be established to create horizontal platforms capable of operability, programmability, and communicability among sensing devices, operating systems, and applications irrespective of model or vendor. Also, interoperability problems can ensue when designing scalable communication frameworks for multiple sensing devices and data centers (cloud servers).

#### 6.5.5. Mobility

Mobility in HIoT systems refers to the ability to leverage network support for patients to connect to the gateway at any time and from any location. The IoT healthcare network should be able to accommodate patients’ mobility and link them at any time and from any location. Current studies of IoT frameworks for patient monitoring continue to ignore the patient’s mobility. In addition, mobility is required to make the network fault-tolerant, allow comprehensive access to information regardless of location, and improve service quality. Since provisioning is critical in healthcare, a mobility protocol should be able to minimize packet losses and network congestion in any situation. As a result, mobility is an intriguing scientific problem.

## 7. Artificial Intelligence

This section discusses the third component (AI) in the taxonomy. In the context of HMS, AI is subdivided into ML and BDA. This classification intends to develop a larger conceptual perspective of AI’s application in HMS. Details on ML, BDA and their respective applications to HMS are presented in the following subsections.

### 7.1. Machine Learning (ML)

In the area of healthcare, ML has a wide technological influence. The value of ML for data analysis in healthcare is becoming prevalent as devices such as wearable sensors are utilized to periodically gather a vast amount of health-related data [118]. Insights derived from these data may be crucial to administering quality healthcare while also assisting in the establishment of a suitable connection between the patient and the doctor. ML can be used in a variety of healthcare applications, such as supporting doctors in identifying more tailored medication and targeted medical processes.

In healthcare, there is a massive quantity of data accessible. This pertains to electronic medical records (EMRs), which may include all forms of data [120]. For instance, structured data refers to data that are simple to categorize in a database; they might contain a set of features and records such as patient’s biodata and generic health complaints such as fever or nausea [121]. On the other hand, some health data are unstructured and are usually in the form of photographs, text files (medical reports and summaries), and audio/visual recordings. Personal conversations, for example, might point in a variety of ways [122].

Realizing and exploiting different forms of data on a large scale would be highly valuable in implementing ML techniques in HMS [121]. Furthermore, when ML is used successfully, it may assist doctors in making near-perfect diagnoses, determining and improving patients’ overall health, and lowering costs [122]. In the healthcare industry, 5% of all patients account for 50% of total expenses; also, the number of chronic disorders requiring ongoing treatment has steadily climbed across the globe. Due to the expensive healthcare services, the application of ML to these health-related data may be the answer to this ever-growing problem. From health-related data, ML can identify people who are more prone to develop common illnesses. Also, most of the hospital visitations are evitable and ML may be utilized to aid in diagnosis and administer suitable therapy. As a result, expenditures are reduced by keeping patients out of expensive emergency rooms [121].

When the training data to be used for an ML process is labelled, a supervised ML technique can be deployed. In the case of unlabeled training data, an unsupervised ML technique can be used while a semi-supervised ML technique can be used when the training data consists of both labelled and unlabeled data. Refs. [123,124] presented a thorough overview of supervised and unsupervised ML techniques.

#### 7.1.1. Supervised Machine Learning

Supervised ML techniques are those that use labelled training data to create relationships or insights from input and output features. A classification process is developed if the output data is discrete and regression if the output data is continuous [125]. Certainly, there is an imperative need for a model that can successfully provide proper output data. However, the presence of noise in the data may lead to an ineffective model. Some of the traditional supervised ML methods include Bayesian network (BN), Decision Tree (DT), and Multi-layer Perceptron (MLP) [126,127]. Instances of supervised ML approaches in healthcare include the categorization of several forms of diseases [128] and the identification of various bodily parts from photographs [129].

#### 7.1.2. Unsupervised Machine Learning

Unsupervised ML methods are ML approaches that use unlabeled data. Unsupervised ML approaches that are often employed include data point clustering using a similarity metric and dimensionality reduction to project high-dimensional data to lower-dimensional subspaces (also known as feature selection) [130]. Exploratory analysis and dimensionality reduction are two typical unsupervised ML applications. Unsupervised ML approaches may be utilized to acquire first insights into data in situations when a human examination is difficult [131]. The findings may be used to test various theories. For dimension reduction, the data is represented by fewer features and unsupervised ML can also be used for this procedure. For that purpose, the link between characteristics must be identified. It may assist us in removing duplicate features. As a result, data processing may be accomplished using a significantly less demanding solution [33]. Unsupervised ML may also be utilized for anomaly identification, such as clustering [132,133]. Prediction of cardiac illnesses using clustering [134] and prediction of hepatitis disease using principal component analysis (PCA), a dimensionality reduction approach [135,136] are two classic instances of unsupervised ML techniques in healthcare.

#### 7.1.3. Semi-Supervised Machine Learning

Semi-supervised ML techniques are beneficial when both labelled and unlabeled samples are available for training, which is often when there is a small quantity of labelled data and a big number of unlabeled data. Since acquiring a significant quantity of labelled data for model training is challenging in healthcare, semi-supervised learning approaches may be very effective for a range of healthcare applications. In the literature, many aspects of semi-supervised ML employing various learning algorithms have been suggested. For example, Fagherazzi, et al. [137] presented a semi-supervised clustering technique for healthcare data, while Yu, et al. [138] suggested a semi-supervised ML strategy for activity detection using sensor data. Peng, et al. [139] and Luo, et al. [140] used a semi-supervised learning strategy to segment medical images.

### 7.2. Application of Sensors and IoT with Machine Learning in HMS

This section examines various existing efforts that combined wearable sensors and IoT with ML techniques in HMS. The review has been divided into sections depending on the kind of ML technique used.

#### 7.2.1. Application of Sensors and IoT with Supervised ML in HMS

Saadatnejad, et al. [141] proposed a new ECG categorization method. This approach was employed on wearable devices to assess heart disease in real-time. The benefit of this technology was its low power usage. They employed wavelet transform and multiple long short-term memory (LSTM) recurrent neural networks (RNN) in their technique. Their strategy produced excellent ECG classification results. Conversely, as reported by Amirshahi and Hashemi [142], a novel ECG classification method was presented and deployed in low-power wearables based on spiking neural networks (SNN). The findings demonstrated its appropriateness for real-time situations. Furthermore, in the real-time categorization of ECG data, its energy usage was much lower than that of other comparable devices. In another study by [143], a classification model was developed to detect irregularities in patients’ breathing sounds. These data were utilized to automate the diagnosis of respiratory and pulmonary diseases. To categorize respiratory sounds, the deep learning (DL) model was deployed. A local log quantization approach was also presented to decrease the memory footprint, which may be employed in memory restricted wearable devices.

Furthermore, sensing devices may be utilized to diagnose diseases using patients’ bodily movements. Hssayeni, et al. [144] employed an LSTM with an RNN to identify early indications of Parkinson’s disease (PD) using accelerometer and gyroscope data. In another work by Ahlrichs, et al. [145], waist-worn accelerators and support vector machines (SVM) were utilized to identify freezing of gait (FoG) in Parkinson’s disease patients. Also, Varatharajan, et al. [146] used a dynamic time warping technique (DWT) and multiple wearable sensors, including accelerometers, to track patients’ walking habits. Early symptoms of Alzheimer’s disease were discovered based on the reported walking style.

#### 7.2.2. Application of Sensors and IoT with Unsupervised ML in HMS

Das, et al. [147] suggested an unsupervised ML technique for HR prediction from ECG data obtained by wearable devices. Spike training was used to directly encode the spatiotemporal features of ECG signals. The spike training was then utilized to stimulate recurrently coupled spiking neurons in a liquid state machine computing model. Using particle swarm optimization (PSO), an unsupervised readout based on fuzzy c-Means clustering of spike responses was created. Their suggested strategy was simple to implement on spiking-based systems. The method’s merits include its excellent accuracy and extremely reduced energy footprint. As a result, the battery life of wearable gadgets increased. Krause, et al. [148] suggested another unsupervised learning approach. An online wearable device was conceived, deployed, and assessed in their study without any outside supervision. It could ascertain the usual user’s context and the likelihood of context transfer. They incorporated statistical analysis and an unsupervised ML technique in their graph algorithm methodologies. The findings demonstrated that their suggested technique could generate a user context model while just requiring data from a device equipped with physiological sensors. Janarthanan, et al. [149] proposed a novel form of unsupervised DL technique that optimized data during preprocessing in wearable sensors. The proposed method took 11.25 ns for its computation with increased performance in its feature selection and feature extraction processes.

#### 7.2.3. Application of Sensors and IoT with Semi-Supervised ML in HMS

Wearable gadgets can capture massive volumes of data. However, classifying this data is expensive and time-consuming. As a result, it is preferable to create strategies for using unlabeled data while minimizing labelling expenses as much as possible. Semi-supervised ML techniques are potential ways for effectively using a combination of limited labelled data and a huge amount of unlabeled data. Ballinger, et al. [150] employed off-the-shelf wearable HR sensors to gather data from many people using a mobile phone application. The goal was to use a multi-task LSTM to identify several medical problems such as diabetes and excessive cholesterol. Two semi-supervised techniques were developed which eventually outperformed hand-engineered biomarkers from the medical literature. In the first technique, an LSTM was pre-trained as a sequence autoencoder. The pre-trained parameters were utilized to kick off a second supervised phase with a pool of restricted labelled data. In the second strategy, the synthesized dataset was utilized for pre-training.

Also, Stikic, et al. [151] proposed a novel approach for activity recognition. Their suggested technique propagated information in a graph that included both labelled and unlabeled data. Two methods for combining several graphs were presented based on feature similarity. In their study, they examined the quality of the label propagation process as well as the performance of classifiers. In another similar study, the possibility of semi-supervised learning to lower the amount of supervision was evaluated for activity recognition [152]. Ma and Ghasemzadeh [153] suggested a human activity identification tagged LabelForest. LabelForest is a non-parametric semi-supervised learning framework for activity identification that enhances the performance of ML algorithms by increasing the training set. LabelForest labels a portion of unlabeled data. The sample is chosen based on its resemblance to the labelled samples. The LabelForest framework is comprised of two algorithms: the spanning forest approach for sample selection and labelling, and the silhouette-based filtering technique for selecting examples with more reliable clustering assignments for inclusion in the training set. Wiechert, et al. [154] recorded EEG brain waves from subjects doing various activities such as reading, listening to music, and so on, using a wearable headgear called Muse. The goal was to identify individuals and the sort of activity they were doing while EEG data were being captured. To do this, K-medoids and an evolutionary algorithm were coupled to produce multiobjective clustering. The genetic algorithm (GA) was employed to discover the best K-medoids. Wiechert, Triff, Liu, Yin, Zhao, Zhong and Lingras [154] reported that their strategy outperformed K-means.

### 7.3. Big Data Analytics (BDA)

Health data detailing patient characteristics and treatment encompasses a wide range of data sources, including text files, imaging, and narrative data, all of which are underutilized and have much more potential to be fulfilled than is now realized [155]. It is commonly acknowledged that applying various data analysis tools to ingest medical data has significant potential for healthcare improvement [67]. The two most common methods for using medical data are information retrieval and data mining. It is important to note that these technologies may be utilized in two ways: first, to collect information for general use, about groups within the studied population, and second, to obtain information on particular people. The two purposes are quite distinct, and implementations of both data-driven technologies vary greatly.

#### 7.3.1. Data Mining in Healthcare

In general, data represents the new gold in the mining metaphor, while analytics systems represent the technology that mines, moulds, and mints it. In practice, HMS is dealing with information overload while caring for patients. Data mining in healthcare can also be referred to as healthcare analytics. Healthcare analytics is described as a collection of computer-based methodologies, processes, and workflows for translating raw health data into useful insights, novel discoveries, and knowledge that can be used to make more effective healthcare decisions [156]. Healthcare analytics has recently gained prominence not just to enhance patient and healthcare services, but also as an efficient cost-cutting tool. Healthcare analytics is gaining prominence because of the Big-Data phenomenon’s emergence in the healthcare arena. In healthcare data, for example, structured data might take the form of electronic medical records (EMRs) or electronic health records (EHRs), which feature conventional input record fields such as patient name, date of birth, address, address, and other field-based information. Instrument readings and data created by the continuing migration of paper records to electronic health and medical records are examples of semi-structured data. Furthermore, structured, and unstructured data streams from fitness gadgets, genetics and genomics, social media, and other sources may cascade into healthcare systems. As a result, healthcare companies are in desperate need of effective methods to integrate and convert such diverse data sets, including automating the translation from unstructured to structured data.

As a result, big data technologies provide a robust framework for extracting relevant information from this sea of data. BDA, according to McKinsey’s research, is the basis for delivering five healthcare values: Right Living, Right Care, Right Provider, Right Value, and Right Innovation [157]. These ideals provide limitless options for enhancing healthcare services while also lowering waste and expenses. Healthcare analytics, for example, can aid in patient classification based not only on simple traditional demographic attributes such as gender, age, and lifestyle, but also on relevant health and clinical characteristics such as medical conditions, risk propensities, genetic disposition, and therapeutic probabilities. Healthcare analytics enable the optimization and tailoring of the course of therapy for each patient based on a plethora of criteria such as past medical history, precautions, allergies, genetic features, personal risk factors, work and lifestyles, and safety management. Healthcare analytics may also reveal causal correlations between a variety of quality metrics and variables that impact or affect those health indicators in a patient group or individual patients. Furthermore, healthcare analytics may be utilized to generate more precise patient risk stratification measures, such as estimating the number of health complications, the influence of comorbidity, and how severe a patient’s health state would affect the result. Risk profiles for patients may be generated using such calculations, which will aid in the construction of treatment regimens for a group of patients with similar profiles. Such categorization would be very beneficial to the planning and pricing of any accountable care organization. These objectives need the availability of strong big data storage, processing, and analytics systems.

#### 7.3.2. Information Retrieval in Healthcare

Information retrieval (IR) is the process of searching vast document collections for the information most relevant to a user’s query, which is about discovering anything or obtaining tiny subsets of documents that are already part of the accessible document sets. It should be noted that for IR, the returned information is precisely the same as it is kept, i.e., there is no value addition to the document sets. The purpose of IR is to get the needed information as quickly and precisely as feasible. In the healthcare arena, IR is primarily concerned with medical text retrieval and medical picture retrieval. Since clinical text concerning diagnostic findings, treatment plans, and patient summaries is the major body of the EMR, it is becoming a vital approach for speedy and efficient access to patient information for medical text search. The medical records track is established in TREC [158]. Medical text retrieval may be seen as a domain-specific text search problem, with the key issue being to cope with the complexity and ambiguity of medical data and queries [159]. Semantic-based text search approaches are widely used to address the ambiguity problem in medical search with the help of standard terminologies or domain ontologies such as the International Classification of Disease (ICD), Unified Medical Language System (UMLS), and Medical Subject Headings (MeSH). Query expansion and reformulation are two mechanisms often used for enhancing search quality. As reported by [160,161], semantic resources are used to describe inquiries in an expressive and meaningful context, therefore filling the semantic gap between queries and EMRs and improving medical search quality. Six existing domain-independent semantic similarity metrics have been extended to the biomedical domain. The medical hierarchy is used to solve the granularity mismatch issue resulting from ambiguous inquiries by describing subsumptions as parent-child connections between ideas [162].

#### 7.3.3. Application of BDA in HMS

In principle, the size of healthcare data is likely to skyrocket in the coming years. In practice, utilizing recent advances in IT to effectively analyze and utilize such big data can result in significant benefits for healthcare organizations. These benefits range from single-doctor offices and multi-provider groups to large hospital networks in a variety of use-cases and application scenarios. Healthcare analytics, as an example, may be used in a variety of applications to convert massive volumes of data into actionable information that can be used to identify needs, deliver services, forecast issues, and avoid crises for the patient population. Among these use-cases are the following:

##### Data-Driven and Evidence-Based Healthcare System

Rather than relying only on intuition, the decision-making process is based on the analysis of large amounts of data that is more indicative of the actual world. For example, the US Healthcare Big Data project has information on nearly 50 million patients [163]. This data is used to uncover difficulties in the healthcare industry, where answering the proper questions for this purpose across such large amounts of data is quite difficult. In addition to clinical data, healthcare data comprises pharmaceutical data (e.g., medication compounds and structures), data on personal behaviours (e.g., exercise routines, eating habits, environmental influences), and billing/financial records. Effectively integrating all of this data is the key to considerable improvements in treatments, delivery, and well-being.

Doctors have typically utilized their discretion when determining treatment choices; however, there has lately been a shift toward evidence-based medicine. This new trend tries to improve decision-making by stressing the use of evidence from well-designed and executed research. This tendency may be supported and realized by collecting and analyzing a range of organized and unstructured health-related data, financial data, and genetic data to match treatments with results, forecast people at risk of developing a disease and deliver more efficient care.

Moreover, Clinical decision support systems are gaining popularity as medical institutions and regulating authorities strive for improved data management to ensure effective and efficient healthcare delivery and excellent results. Analytics methods may be used to analyze massive volumes of data, interpret, categorize, and learn from this data to forecast results or offer alternative therapies to doctors and patients at the point of care. Furthermore, comparative effectiveness research may be used to identify more clinically relevant and cost-effective methods of diagnosing and treating patients.

##### Healthcare Patient and Unstructured Data Profiling

This entails using sophisticated analytics to patient profiles to identify patients who might benefit from proactive therapy or lifestyle adjustments. Applying predictive modelling methods, for example, to model and identify the profiles of patients who are at risk of acquiring a certain illness (e.g., diabetes) and should be exposed to preventative therapy. In addition, According to Gartner and IBM52, the majority of data (80%) now resides in unstructured or semi-structured formats, from which a lot of information may be gathered [59]. In healthcare systems, clinical notes include a wealth of information that is difficult to extract. Furthermore, medical workers and specialists must stay up with medical literature. Big data processing technologies may be efficiently used to find highly relevant data and literature from unstructured language and turn it into readily processable data.

##### Efficient Healthcare Policy

This type of situation entails using analytics methods on illness patterns to detect disease outbreaks and transmission which can be used to design prompt and effective public health monitoring systems. It may also be used for the development of targeted immunization strategies that are more efficient and effective. Furthermore, a BDA system may be used to capture as well as analyze social media data to forecast disease outbreaks based on search, social content, and query behaviours of users. Many researchers, for example, are presently utilizing Google Trends services to investigate the date and location of search engine inquiries to forecast illness outbreaks [164].

##### Genomic Analytics

Recently, the efficiency of the gene sequencing process has been dramatically enhanced, and the cost has been significantly reduced. The 1000 Genomes Project 51, for example, has been launched as a multinational research endeavour managed by a coalition of 75 corporations and organizations to create the most comprehensive record of human genetic diversity. The initiative has expanded to 200 terabytes of genetic data for over 1700 people, which researchers may now freely access and analyze using Amazon Web Services for disease research. Thus, efficient genomic analytics techniques can make the genomic analysis process to be a main component of the regular medical care decision process and the growing patient medical record [165]. Genomic analytics may help find the links between a disease and its genetic, environmental, and/or health-related risk factors. This study may provide a unique insight into the underlying processes of illnesses and disorders, as well as disclose the interaction of several kinds of risk factors. Identifying risk-based genes is an important step toward discovering molecular pathways for direct treatments, while personal risk factors create corrective measures that patients may use to lower their chances of getting certain illnesses.

As a result, effective tailored care services based on DNA sequence information may be used in real-time to highlight best practice therapies for patients. This allows for a shift away from a population-level epidemiological approach and toward tiny groups or people characterized by their biochemistry and genetics. Furthermore, this contributes to the continuing and progressive transition from disease-centered to patient-centered treatment. In this scenario, a single patient may have terabytes of data in multiple formats, including huge and complicated genetic, proteinic, and metabolic data. NoSQL technology is capable of effectively coping with such complicated and huge datasets.

##### Enhanced Remote and Evidential Healthcare Delivery

Sensing technologies are helping to improve the process of obtaining and evaluating real-time and fast-moving patient data from in-hospital and at-home devices. Real-time analysis of such data may considerably enhance patient safety monitoring and the accuracy of the event prediction process. Furthermore, knowing how to discover recurring patterns in signals received from sensing devices might be useful in recommending a non-invasive method of learning underlying physiological processes. Big streaming processing systems may play a significant part in realizing the sorts of applications that can be designed to meet these circumstances.

##### Social Media of Healthcare

PatientsLikeMe is an example of a patient social network that began in 2006 and today includes over 200,000 patients and tracks 1500 illnesses. People may connect with others who have the same ailment or condition, monitor, and share their own experiences, learn what therapies have benefited other patients like themselves, acquire insights, and uncover any common trends on such a platform. Furthermore, patients contribute continuing data on their unique illnesses, treatment history, side effects, hospitalizations, symptoms, disease-specific functional scores, weight, mood, quality of life, and other factors. As the need for obtaining and processing health-related information from social networks grows, big data analytic tools may play a crucial role in digesting and evaluating such expanding datasets.

### 7.4. Challenges and Open Issues of AI in HMS

Despite the positive outcomes obtained using ML and BDA techniques in HMS, there are still unresolved obstacles in clinical healthcare application. The following are the primary concerns and challenges:

#### 7.4.1. Size, Quality and Temporality of Data

ML refers to a set of incredibly complex computing models, such as neural networks, that are entirely linked multi-layer and need many parameters to be estimated properly. It should supply a massive quantity of data to achieve this goal. Furthermore, comprehending diseases and their unpredictability is more difficult than other activities. For example, voice pathology type detection via speech and image processing in Magnetic Resonance Imaging (MRI) can predict Alzheimer’s sickness. As a result, from the standpoint of BDA, it is critical to have a larger quantity of medical data to build a strong and effective ML model. In addition, healthcare data is highly varied, incomplete, and unclear. Training an effective ML model with such a diverse and massive amount of data is tough, and several factors must be considered, such as data sparsity, missing values, and redundancy. The ailments are continually changing and developing throughout time. Several suggested machine learning models in various healthcare sectors, however, assume static vector-based inputs. These static inputs couldn’t handle the time factor. A new ML approach that can cope with temporal medical data is an important feature that will need to be created. In other words, it is critical to design novel ML techniques and frameworks that consider dynamic inputs.

#### 7.4.2. Field Complexity

Healthcare applications and biomedicine are growing more complicated. The ailments are very varied, and most of them, their progression, and their causes are still unknown. Furthermore, the number of patients in a practical clinical is frequently restricted, thus we cannot request as many patients as we would want. As a result, medical training data are scarce for an efficient ML model.

#### 7.4.3. Ethics and Policy Issues

Establishing and maintaining data ethics in user-centric ML and BDA applications such as in HMS is imperative. Before gathering data for creating ML models, explicit efforts should be made to understand the intended user community and their social features. Also, recognizing how data collection might jeopardize a patient’s well-being and dignity is critical in this context. If ethical problems are ignored, the use of ML and BDA in practical contexts will have negative consequences. Furthermore, it is critical to have a comprehensive knowledge of the AI system in unpredictable and complicated settings to guarantee the fair and ethical functioning of automated systems [166]. Concerning policy problems, only by resolving regulatory and legislative constraints can the full potential of ML and BDA systems (which effectively comprise software as a medical device) in actual healthcare settings be realized. According to the research, regulatory criteria are required for both medical ML systems and their integration in real clinical contexts [167]. As a result, the integration of AI-powered ML and BDA systems in the real clinical setting must adhere to the norms and regulations established by the government and regulatory authorities. Existing regulations, however, are insufficient for certifying systems that are constantly evolving, such as ML-enabled systems, because another key challenge with the use of ML algorithms in clinical practice is determining how these models should be implemented and regulated, given that these models will incorporate learning from new patient data [36]. Furthermore, objective clinical assessment of ML systems for specific clinical contexts is critical to ensuring safe, effective, and robust functioning that does not damage patients in any manner. Data scientists and AI engineers should be hired in hospitals to evaluate AI systems regularly to verify that they are still safe, relevant, and functional.

#### 7.4.4. Safety and Privacy Issues

Success in a regulated laboratory environment (a frequent ML community practice) is not proof of safety. The safety of ML and BDA is the assessment of how safe the implemented system is for patients. Throughout ML’s lifetime, safety should be a continual focus. Many regular physician jobs are routine, and the patients they meet have common health issues. It is their job to diagnose uncommon, subtle and concealed health disorders that arise once in a million. Enabling ML to perform effectively on buried strata, outliers, edges, and delicate instances is critical to ensuring the safety of contemporary AI systems. In addition, one of the primary difficulties in data-driven healthcare is privacy, which is concerned with the usage of users’ data by ML systems for prediction. Users (in this case patients) expect their healthcare providers to take the required precautions to protect their inherent right to the privacy of their personal information, such as age, gender, date of birth, and health data. Potential privacy hazards might be of two types: revealing sensitive information and harmful data usage (potentially by unauthorized agents). The qualities and type of the data being gathered, the context in which it was produced, and the demographics of the patients all influence privacy. As a result, mitigating privacy breaches with the proper technique(s) is crucial, as such breaches may directly affect patients. To avoid privacy violations such as (re-)identification of persons, sensitive data should be anonymized [168]. Furthermore, enough attention should be made to understanding privacy issues at each level of data processing, and data transmission across departments within a hospital should be conveyed in a secure environment. Adoption of ML techniques for building biometric healthcare systems either offline or online raises privacy concerns. Since the security and privacy of such systems are critical, a worst-case robustness test for biometrically secure healthcare systems should be done. Worst-case testing is a strong method that can offer adequate information regarding a system’s resilience and discriminate between a system that never fails and a system that fails once in billion trials.

#### 7.4.5. Causality Problem

Recognizing causality as a problem in healthcare is critical since the majority of critical healthcare issues need causal reasoning [36]. For example, ask what would happen if a doctor recommended therapy A instead of treatment B. Such problems cannot be answered using conventional ML algorithms, and we must examine the data from the perspective of causality [169]. In healthcare, learning is often based exclusively on observational data, and addressing causal questions through learning from observational data is difficult, necessitating the development of causal models. Some ML models (ensemble and DL methods) are black boxes with no core underlying theory, and they primarily function by exploiting patterns and correlations without taking into account any causal relationship [170]. In general, this is not a constraint since prediction does not need any causal relationship. In BDA, the lack of a causal relationship raises concerns regarding the implications that may be derived from ML model findings. Furthermore, via the lens of causal reasoning, fairness in decision-making may be effectively enforced [171,172]. To provide fair predictions, the causal influence of some variable(s) on a target output (e.g., target class in a multi-class classification issue) must be estimated.

## 8. Blockchain

**Figure 8:**
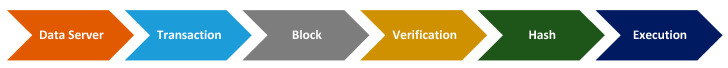
Fundamental Functioning Stages of Blockchain Technology.

Blockchain is a network of decentralized nodes that holds data. It is a great solution for safeguarding sensitive data inside the system. This technology facilitates the flow of essential data while keeping it private and secret. It is an ideal solution for securely storing all linked papers in one area [173,174,175]. Using a single patient database, Blockchain also speeds up searches for candidates who meet certain trial requirements. The Blockchain is a peer-to-peer (P2P) network of personal computers known as nodes that maintain, save, and record history or transaction data [176,177,178]. It enables dependable cooperation since information is kept and distributed by all network users, and it maintains track of previous and present experiences. This technology can connect diverse networks and give insights into the significance of individual therapy. As a result, Blockchain may be recognized for its immutability and security. Blockchain’s three primary concepts are blocks, nodes, and miners. Blockchain does not save any of its data in a single place. Instead, the Blockchain is replicated and propagated by a network of computers. Every computer on the Internet updates its Blockchain to reflect the addition of a new block to the Blockchain. Figure 8 depicts the fundamental functioning stages of Blockchain technology.

### 8.1. Need for Blockchain in Healthcare

The necessity for improvement in the sphere of healthcare is expanding at an alarming rate. Nowadays, there is a demand for strong healthcare facilities that are supported by cutting-edge technology. In this situation, Blockchain would play a significant role in revolutionizing the healthcare industry. Additionally, the healthcare environment is evolving toward a patient-centered approach that emphasizes two critical aspects: always-accessible services and enough healthcare resources. Blockchain technology can improve the conventional HMS in terms of prompt patient care and healthcare facilities. Another time-consuming and repetitive activity that contributes to high healthcare expenses may be resolved swiftly with Blockchain technology. Patients may participate in health research initiatives using Blockchain technology. Furthermore, informed decisions and data sharing on public well-being would improve therapy for most people [176,178,179,180].

To date, the prominent issues in HMS have been data privacy, data sharing, and interoperability. The incorporation of Blockchain with HMS can address these lingering problems.

### 8.2. Application of Blockchain in Healthcare

Blockchain is a relatively new and rising technology with creative possibilities in healthcare implementation. Smooth, fast data exchange and delivery among all significant network members and healthcare providers contribute to the development of cost-effective medications and advanced treatments for a wide range of ailments. This will hasten healthcare expansion in the future years. The benefits of Blockchain technology in the transportation business have lately been shown, as have the benefits of Blockchain technology in the healthcare sector. Since this field has a direct impact on the quality of living, it is one of the first areas where digital transformation improves, and innovations occur. Simultaneously, Blockchain technology is gaining popularity, particularly in the financial sector. It provides various significant and amazing opportunities for the healthcare business, ranging from science and logistics to interactions between health workers and patients [180,181,182]. Table 5 summarizes the key important Blockchain applications in healthcare.

Aside from conventional HMS frameworks, the purpose of Blockchain is to document all types of transactions in a decentralized record. It is precise and simple, thereby reducing managerial effort [178,180]. Blockchain’s use in health care seems to be very promising and exciting since it helps to resolve some of the industry’s most urgent concerns. Blockchain can be used to link other services onto the network, allowing everyone to access the same data. By using Blockchain technology in health care, several techniques will bring great outcomes for organizations. It delivers patient information, medical research, clinical trials, the medical supply chain, and product integrity [176,177,182,197].

### 8.3. Use-Cases of Blockchain in HMS

In support of the preceding section (Section 8.2), this section depicts many use-cases of Blockchain technology in HMS for achieving various advantages in the healthcare sector. Specifically, Blockchain properties such as decentralization, enhanced data security and privacy [180], data ownership, availability and resilience, openness and trust, and data verifiability are explored.

#### 8.3.1. A seamless HMS

One of the most serious health issues nowadays is the fact that health organizations maintain several fragmented health data on their patients. This issue may be addressed by incorporating Blockchain technology in medical record transfers [182]. To provide restricted access to a patient’s electronic health record, a smart contract may be created to construct a smart health ecosystem. Doctors will make notes, insert scans, and administer tests, all of which will be recorded as transactions. When a pharmacy provides medicine, the transaction is recorded on the Blockchain. The patient grants his or her insurer limited access to the patient’s check treatment and payment records. Doctors will be able to remotely assess medical problems and provide advice or a second opinion. Health insurers will provide tokens or decrease rates to patients to measure their development and fitness. Some tokens may be produced, which patients will use on the Blockchain to store electronic health information and transfer data from wearable health devices. This Blockchain-based healthcare ecosystem will enable developers to create smart applications that analyze health data and provide suggestions on health issues.

#### 8.3.2. Electronic Health Record (EHR)

Over the years, the health industry has been impacted by the development of centralized systems, the regulation of health data, and the obligation to collaborate with multiple EHR service providers to digitize medical data [197]. Most repositories do not communicate with one another, but they include information held by health professionals, pharmaceutical corporations, and other health ecosystem stakeholders. The difficulty of most quantitative health data systems to communicate with one another, both at the individual (patient) and community (public) levels, exemplifies the structural issues that are often encountered in the following situations:

- When patients desire other healthcare practitioners to contact them or seek medical treatments on their behalf.
- When clinical trial administrators want to authenticate the vast medical data of their participants.
- When pharmaceutical corporations want to assure that pharmaceuticals distributed on worldwide marketplaces are genuine.

Blockchain technology has the potential to increase healthcare professionals’ and the whole healthcare industry’s performance, patient data openness, monitoring, and accountability, while also lowering costs. Furthermore, a variety of Blockchain solutions may be adapted to meet diverse healthcare applications.

#### 8.3.3. Healthcare Payments

The pre-authorization procedure, which some insurance companies need before agreeing to pay for certain treatments, is today’s significant source of frustration. Determining whether a particular item is covered by a member’s insurance policy may be a time-consuming procedure for a variety of reasons, including the participation of various stakeholders in the difference in the amount covered. The procedures are often complicated, entail multiple manual stages, and might be the result of inadequate communication and technology, depending on the payer-provider relationship. With Blockchain, a single ledger maintained by healthcare stakeholders holds the patient’s entitlement as well as smart contracts that encode the pre-authorization requirements. This automates information gathering and dissemination, enabling benefits to be calculated in real-time. Some of the benefits of adopting Blockchain for pre-authorization include increased cash flow owing to quicker transaction settlement, prompt patient care, correct payment to the provider, lower administrative expenses, and double record-keeping.

All parties in the claim specifications and reimbursement criteria are held accountable in a Blockchain-enabled HMS. Smart contracts can be used to negotiate and hold contracts between suppliers, clients, payers, and governmental regulators. Before submitting claims, healthcare practitioners would know precisely what information is required. Providers may simply format claim data to guarantee that all data is input properly and accurately, as needed by the Blockchain. This clarity decreases or eliminates the number of claims that are returned due to inadequate information, saving time and effort for all parties involved. Blockchain technology is gaining traction in healthcare, and it may benefit every stakeholder in the health sector.

To be ready for tomorrow’s transformations, health organizations must recognize and test Blockchain technology today.

#### 8.3.4. Pharmaceutical Supply Chains

Safety, stability, and security are among the highest criteria in the pharmaceutical sector. Blockchain technology, for example, can be used to monitor supply chain management securely and transparently. This may greatly decrease delays and human mistakes. It may also be used to monitor costs, labour, and even waste pollution at every point in the supply chain [198]. They may also be used to check product validity by tracking their origin and combating the counterfeit medication industry, which costs $200 billion in losses each year. Companies such as Chronicled, Blockpharma, and Modum are already developing more efficient Blockchain logistic solutions. Modum, as an example, operates following EU standards that demand verification that pharmaceutical items have not been subjected to specified circumstances, particularly high temperatures, that might jeopardize their purity. Modum’s answer was to create a sensor that collects environmental variables while actual objects move and stores them permanently on the Blockchain [180].

### 8.4. Challenges and Open Issues of Blockchain in Healthcare

Although Satoshi Nakamoto pioneered Blockchain technology in 2008 [199], it is still in its infancy. It was designed to function as a public transaction ledger for the Bitcoin cryptocurrency. Since 2008, there has been a dearth of serious studies, and not enough emphasis has been placed on the actual technology behind Bitcoin. This lack of study has an impact on the adoption of Blockchain technology. There is no legal structure to support or support smart contracts, making it more difficult for enterprises to fully adopt and integrate them into their operations.

Deloitte conducted a Global Blockchain Survey in early 2019 (Deloitte’s 2019 Global Blockchain Survey), and 29% of respondents highlighted a lack of knowledge of Blockchain technology as one of the major barriers to adoption [180,200]. Research has continued to focus on Bitcoin rather than the infrastructure that Bitcoin employs. There is inadequate proof of Blockchain technology’s genuine usefulness, and 22% of respondents to the Deloitte Global Blockchain Survey concur. The following factors as listed in the succeeding subsections may contribute to apprehension about using Blockchain technology in healthcare.

#### 8.4.1. Cost of Acceptance and Adoption

There are currently not many systems that are built to make use of Blockchain technology wholistically. When Blockchain technology is intended to be used for a particular purpose, developers must write their code (or hire someone else to do it)—creating new software costs a lot of money, ranging from $10,000 for simple apps to $800,000 or more for unique software with complicated features.

#### 8.4.2. Limited Blockchain Experts

Adoption is being hampered by a scarcity of specialists in two ways. For starters, there aren’t enough individuals working on innovation to keep up with demand. Second, exclusivity implies that existing Blockchain professionals are expensive to hire. In an ideal world, increased interest in Blockchain technology would drive more individuals to learn more about it.

#### 8.4.3. Regulations, Policies and Government

Regulation, policies and willingness of Government are major barriers to Blockchain technology adoption, despite the technology’s promise [201]. Deloitte performed research in 2019 in which managers were asked, “What are the impediments to increasing the usage and scope of Blockchain technology in your firm or strategy if any?” [180]. According to 30% of respondents, regulatory issues alongside implementation problems are the primary impediments to additional Blockchain investment.

Perhaps the technology’s embryonic nature, or its affiliation with Bitcoin, has made many companies wary. Concerns that a distributed computerized system is fully dependent may also emerge, preventing the Blockchain from being used to tackle complicated issues such as DSCSA compliance (Drug Supply Chain Security Act). The deployment of Blockchain requires trust in data repositories without relying on a trustworthy broker to validate a transaction, which prevents healthcare organizations from storing and transferring sensitive information in the HMS.

## 9. Open Research Problems on Emerging Technologies in Healthcare Delivery

Many critical efforts have already been made by researchers around IoT-based applications in the healthcare sector utilizing AI; nevertheless, some constraints remain, which are highlighted in the outstanding problems. As the concepts of IoT and next-generation ML (i.e., deep learning and reinforcement learning) are growing, numerous new paths for exploration will be created. As a result, research problems and open topics have been found during this study and are discussed in the following subsections.

### 9.1. Data Acquisition

Well-curated datasets are critical to the success of AI-based HMS. Several efforts have been made to train Supervised ML techniques using real-time data [16,28]. However, most of the research utilized datasets acquired from public sources supplied by third-party individuals or organizations. These datasets often include unintended bias, lack of variation and missing values. Dataset gathering is a critical undertaking, particularly in the healthcare industry. Furthermore, changes in the environment’s control factors may alter the nature and efficacy of the dataset. Also, the data held in HMS are often incompatible, posing an additional degree of difficulty.

### 9.2. Handling Data Streams

Data Stream Mining is a technique for extracting usable information from continuous and rapid data streams. The modern healthcare sector employs real-time components to gather immediate valuable physiological information with the introduction of remote HMS. This notion addresses how to handle continuous and large data streams [202]. The data that flows via the IoT framework from sensors has a high volume, velocity, diversity, and validity [203,204]. The difficulty is therefore in storing and processing this stream of data, managing difficulties such as concept drift, model retraining, imbalance class labels, high dimensionality, adaptive model selection, and so on. Overall, this is a novel field that ought to be explored more in the context of IoT-based HMS.

### 9.3. Security

Typically, health-related data are acquired from patients (actively or passively), transmitted, and stored in repositories, which are critical in receiving the appropriate treatment or attention. As a result, it is imperative to ensure the security of the patient’s sensitive data. During the data transport, processing, gathering, and storage stages, security refers to the protection of data against unauthorized access or change [205]. Aside from that, various precautions should be taken at the sensor-closest level to secure user data. To offer data security, AI-based HMS may be a preferable alternative [34]. The following three critical security characteristics must be guaranteed [205]:

- Confidentiality: Encryption is the proper technology for ensuring data confidentiality, which necessitates the distribution of a shared key over a WSN communication channel [34].
- Integrity: An attacker should not be able to modify the health-related data contained inside a device. To ensure the accuracy and consistency of the information, the required precautions must be taken.
- Availability: A patient’s health data should be available immediately when requested by an authorized party. Patients’ data is sensitive, and it should be always accessible and available from the network.

### 9.4. Privacy and Ethics

Privacy refers to how the issuing authority handles a patient’s data [205]. It should also specify where the data will be stored and who will have access to a patient’s medical data. In general, doctors, nurses, and caregivers may access and handle this data. However, in certain emergencies, this sensitive data must be shared with other individuals as well to obtain proper treatment or attention [21]. Furthermore, patients may be reluctant to divulge too much personal data (for example, an early pregnancy) to anybody other than family members. As a result, such data should not be made public. Furthermore, since the sensing layer is closest to the user, it requires additional protection from external threats [206]. Personal data on a patient is kept in the storage unit during remote monitoring. As a result, any illegal access to such personal data must be protected [207,208]. It should be noted that there must be a trade-off between maintaining the privacy and offering tailored service. With the increment in health-related data, ethical concerns about the decision-making capabilities of AI-HMS are becoming more pressing. The predictability of AI models is often overestimated, and the techniques lack empathy, which is a critical problem in the healthcare sector.

### 9.5. Explainable AI

There has been a phenomenal rise in supervised ML utilizing multi-modal DL in recent ML studies. Decisions in the healthcare sector are often based on comparable historical facts/data. However, this has opened a new vista in the AI domain and warrants more research in the healthcare sector [21]. In addition, ML models such as Generative Adversarial Networks (GANs) are increasingly often employed in dataset augmentation, boosting the overall performance of ML algorithms. GANs are, to the best of our knowledge, underutilized in this field [209,210]. Deep Reinforcement Learning-based approaches have recently been used in agent-based modelling in the health care area. However, a more advanced design of the HMS may be required before RL-based algorithms can be widely used.

### 9.6. Underdeveloped Countries

It was observed in the literature that researchers often make assumptions that are dependent on the infrastructure of the research area or locale. The successful reported AI models are mostly from developed nations. On the other hand, relatively few successful works have been carried out in underdeveloped regions [21]. Lack of healthcare infrastructure may be the primary barrier same as lack of a communication network (such as the Internet), limited access to data, higher device costs, and hostile and severe laws that are an encumbrance to the healthcare sector.

## 10. Conclusions

In recent years, Sensors, IoT, AI and Blockchain have been widely deployed in many areas. These emerging technologies are being deployed in healthcare for the enhancement of HMS. Therefore, researchers are paying close attention to the deployment of these technologies in healthcare. In this research, a survey was conducted to identify the application, challenges, and open research areas of Sensor-IoT-AI-Blockchain-based HMS. Specifically, a unique taxonomy that illustrates the whole process of Sensor-IoT-AI-Blockchain-based HMS is proposed. For convenience, the whole process is separated into four major areas: Sensors, IoT, AI, and Blockchain. Data collection and transmission are accomplished with sensors and IoT frameworks, while AI and Blockchain allow intelligent decision-making in healthcare systems. Various aspects of this process have been explored throughout this survey.

From the reviewed literature, it was observed that Sensors and IoT frameworks have been successfully deployed in several HMS. In particular, sensors and IoT technologies have effectively improved HMS operations by enabling efficient and smart diagnosis, supervision, and treatment of diseases and ailments. Nonetheless, it was observed that despite the successful implementations of sensors and IoT in HMS, some critical open issues such as user acceptance, data synchronization, scalability, and interoperability of sensing and IoT devices, data security and privacy, and streamlining practices must be addressed.

In addition, AI and Blockchain technologies are becoming more relevant to the healthcare domain. Specifically, the AI and Blockchain technologies offer platforms for data storage, data visualization and analysis that are quite essential for efficient HMS. AI provides advanced methods that can generate actionable insights and knowledge from health-related data that can be used for immediate and future reference in HMS. Also, Blockchain can provide platforms for health professionals to create decentralized data storage for health-related data that only authorized parties can access to address the issue of data theft and security. However, it was observed that data quality, ethics and privacy issues are some of the barriers to AI in HMS while implementation (cost and user acceptance) and regulatory policies are the key factors against the deployment of Blockchain in HMS.

Consequently, the key value of this study is that it provides readers with a detailed review of Sensor-IoT-AI-Blockchain-based HMS research. Another innovative aspect of the study is the collection of research works based on all the taxonomy’s tiers and sub-tiers and assessed them from the standpoint of healthcare. This method of presenting is distinct from standard survey works.

We are certain that our effort will assist both academic and industrial researchers. A researcher may obtain appropriate guidance on various kinds of sensors that may be employed for different kinds of diseases. Researchers may also learn about the technical details of the Sensor-IoT-AI-Blockchain-based HMS. Furthermore, the open research challenges addressed in this study provide the researchers with some obvious future research possibilities.

## Figures and Tables

**Table 1:** Comparison of this survey with existing related survey papers.

Year	Reference	Taxonomy	Sensors	IoT	Artificial Intelligence	Blockchain	Covered Year
2015	Islam, Kwak, Kabir, Hossain and Kwak [65]	No	No	Yes	No	No	Not stated
2015	Li, Lu and McDonald-Maier [66]	No	Yes	No	No	No	Not stated
2015	Yang, Li, Mulder, Wang, Chen, Wu, Wang and Pan [67]	No	Yes	No	No	No	Not stated
2015	Wahaishi, Samani and Ghenniwa [68]	No	No	Yes	No	No	Not stated
2016	Yeole and Kalbande [63]	No	No	Yes	No	No	Not stated
2016	Yuehong, Zeng, Chen and Fan [56]	No	No	Yes	No	No	Not stated
2016	Capraro [57]	No	No	Yes	Yes	No	Not stated
2016	Azzawi, Hassan and Bakar [58]	No	No	Yes	Yes	No	Not stated
2016	Sakr and Elgammal [59]	No	No	No	Yes	No	Not stated
2016	Romero, Chatterjee and Armentano [61]	No	Yes	Yes	Yes	No	Not stated
2016	Mathew and Pillai [62]	No	No	No	Yes	No	Not stated
2016	Dimitrievski, Zdravevski, Lameski and Trajkovik [64]	No	No	Yes	No	No	Not stated
2016	Hossain and Muhammad [60]	No	No	Yes	No	No	Not stated
2017	Sethi and Sarangi [53]	Yes	No	Yes	No	No	Not Stated
2017	Qi, Yang, Min, Amft, Dong and Xu [54]	No	Yes	Yes	Yes	No	Not Stated
2017	Farahani, Firouzi, Chang, Badaroglu, Constant and Mankodiya [51]	No	No	Yes	No	No	Not Stated
2017	Darwish, Hassanien, Elhoseny, Sangaiah and Muhammad [52]	No	No	Yes	No	No	Not Stated
2017	Tokognon, Gao, Tian and Yan [55]	No	No	Yes	No	No	Not Stated
2018	Cui, Yang, Chen, Ming, Lu and Qin [46]	No	No	Yes	Yes	No	Not Stated
2018	Alam, Malik, Khan, Pardy, Kuusik and Le Moullec [47]	No	No	Yes	No	No	Not Stated
2018	Sharma and Singh [48]	No	No	Yes	Yes	No	Not Stated
2018	Babu and Shantharajah [49]	No	No	Yes	Yes	No	Not Stated
2018	Sughasiny and Rajeshwari [50]	No	No	No	Yes	No	Not Stated
2019	Mutlag, Abd Ghani, Arunkumar, Mohammed and Mohd [43]	Yes	No	Yes	No	No	2007–2017
2019	Habibzadeh, Dinesh, Shishvan, Boggio-Dandry, Sharma and Soyata [42]	No	Yes	Yes	No	No	Not Stated
2019	Dang, Piran, Han, Min and Moon [45]	No	No	Yes	No	No	2015–2019
2019	Dhanvijay and Patil [41]	No	No	Yes	No	No	Not Stated
2019	Ray, Dash and De [44]	Yes	No	Yes	No	No	Not Stated
2020	Santos, Munoz, Olivares, Rebouças Filho, Del Ser and de Albuquerque [38]	No	No	Yes	Yes	No	2015–2028
2020	Amin and Hossain [39]	Yes	No	Yes	Yes	Yes	Not Stated
2020	Alshehri and Muhammad [40]	Yes	No	Yes	Yes	No	2014–2020
2020	Qadri, Nauman, Zikria, Vasilakos and Kim [34]	No	No	Yes	Yes	Yes	Not Stated
2020	Karthick and Pankajavalli [37]	Yes	Yes	Yes	No	No	Not Stated
2020	Al-Dhief, Latiff, Malik, Salim, Baki, Albadr and Mohammed [35]	No	No	Yes	Yes	No	Not Stated
2020	Qayyum, Qadir, Bilal and Al-Fuqaha [36]	No	No	No	Yes	No	Not Stated
2021	Krishnamoorthy, Dua and Gupta [30]	Yes	No	Yes	No	No	Not Stated
2021	Li, Chai, Khan, Jan, Verma, Menon and Li [31]	Yes	No	Yes	Yes	No	2016–2020
2021	Sworna, Islam, Shatabda and Islam [21]	Yes	Yes	Yes	Yes	No	Not Stated
2021	Tunc, Gures and Shayea [32]	No	No	Yes	Yes	Yes	Not Stated
2021	Nahavandi, Alizadehsani, Khosravi and Acharya [33]	No	Yes	No	Yes	No	Not Stated
2022	Kamruzzaman, Alrashdi and Alqazzaz [26]	No	No	Yes	Yes	No	2016–2021
2022	Yang, Wang, Jiang, Guo, Cheng and Chen [27]	No	No	Yes	No	No	Not Stated
2022	Karatas, Eriskin, Deveci, Pamucar and Garg [28]	No	No	No	Yes	No	Not Stated
2022	Alshamrani [29]	No	No	Yes	Yes	No	Not Stated
2022	This study	Yes	Yes	Yes	Yes	Yes	2015–2022

**Table 2:** Categorization of Wearable Sensors.

Type of Sensor	Subcategories	Examples
Wearable sensors	Inertial sensors	Accelerometer
		Gyroscopes
		Pressure sensors
		Magnetic field sensors
	Location sensors	Global Positioning System (GPS)
		Blood pressure cuff
		Electrocardiogram (ECG)
	Physiological sensors	Spirometer
		Esophagogastroduodenoscopy (EDG)
		Galvanic Skin Response (GSR)
	Image sensors	SenseCam

**Table 3:** Categorization of Ambient Sensors.

Type of Sensor	Subcategories	Examples
Ambient sensors	Environmental sensors	Thermometer
		Hygrometer
	Binary sensors	Window contact
		Door contact
		Light switch
		Remote control switch
	Location sensors	Infra-red
	Physiological sensors	Zigbee
		Active Radio Frequency Identification (RFID)
	Tags	RFID tags
		Near Field Communication (NFC) tags

**Table 4:** Comparison of different development boards.

Development Boards	Random Access Memory (RAM)	Operating System	Micro Controller	Processor Speed
Arduino	2 KB	Windows, macOS and Linux	At-Mega328p MC	16 MHz
Beagle Bone	512 MB	Linux and Debian	ARM Cortex A8 32bits	1 GHz
Raspberry Pi	1 GB	Linux, Debian, Android, Windows, etc.	Raspberry Pi Pico RP 2040	1.2 GHz
Intel Edison	4 GB	Windows, macOS and Linux	Intel Quark	500 MHz
Banana Pi	1 GB	OpenWRT and Android, Lubuntu, Ubuntu, Debian, and Raspbian	ARM Cortex A55 CPU	1.8 GHz
Jetson Nano	4 GB	Linux4Tegra	Quad-core ARM Cortex-A57 MPCore processor	1.43 GHz

**Table 5:** Summary of key Blockchain applications in healthcare.

S/No.	Blockchain Applications	Summary	References
1.	Patient’s data storage	The patient’s biodata and medical history are recorded in EHR format by the healthcare provider which can be stored on blockchain-enable platforms. In this case, healthcare providers can traverse the stored data and check for validity seamlessly by regularly matching health records stored on the Blockchain system. Besides, Blockchain provides cryptographic methods, which can be useful in the safeguarding of data and data sharing.	[174,183,184]
2.	Data Validation	Deployment of blockchain can adequately validate data at any point. All transactions are algorithmically validated and linked together in a Blockchain system.	[185,186,187]
3.	Smooth and transparent data manipulation	Blockchain can provide a smooth data exchange among health providers that could enhance diagnostic procedures and precision. Blockchain allows multiple HMS to stay in contact and exchange data on a shared distributed ledger for improved security and accountability.	[188,189,190]
4.	Overhead cost and time reduction	Blockchain systems can easily address the interoperability issue, of missing data in healthcare systems. Health providers will have overview access to patients’ records without the need for third-party applications. This invariably minimizes the cost and time of data transformation.	[191,192,193]
5.	Patient Monitoring	Blockchain may be used with IoT technology to increase the adaptability and integrity of the supply chain, making healthcare logistics increasingly accessible for effective healthcare management.	[194,195,196]
